# Computer simulation of molecular recognition in biomolecular system: from in silico screening to generalized ensembles

**DOI:** 10.1007/s12551-022-01015-8

**Published:** 2022-11-28

**Authors:** Yoshifumi Fukunishi, Junichi Higo, Kota Kasahara

**Affiliations:** 1 Cellular and Molecular Biotechnology Research Institute, National Institute of Advanced Industrial Science and Technology (AIST), 2-3-26, Aomi, Koto-Ku, Tokyo, 135-0064 Japan; 2 Graduate School of Information Science, University of Hyogo, 7-1-28 Minatojima Minamimachi, Chuo-Ku, Kobe, Hyogo 650-0047 Japan; 3 Research Organization of Science and Technology, Ritsumeikan University, 1-1-1 Noji-Higashi, Kusatsu, Shiga 525-8577 Japan; 4 College of Life Sciences, Ritsumeikan University, 1-1-1 Noji-Higashi, Kusatsu, Shiga 525-8577 Japan

**Keywords:** Free-energy landscape, Energy basin, Molecular binding, Conformation sampling, Thermodynamic integration, Weighted ensemble analysis method, Enhanced sampling, Drug discovery

## Abstract

Prediction of ligand-receptor complex structure is important in both the basic science and the industry such as drug discovery. We report various computation molecular docking methods: fundamental in silico (virtual) screening, ensemble docking, enhanced sampling (generalized ensemble) methods, and other methods to improve the accuracy of the complex structure. We explain not only the merits of these methods but also their limits of application and discuss some interaction terms which are not considered in the in silico methods. In silico screening and ensemble docking are useful when one focuses on obtaining the native complex structure (the most thermodynamically stable complex). Generalized ensemble method provides a free-energy landscape, which shows the distribution of the most stable complex structure and semi-stable ones in a conformational space. Also, barriers separating those stable structures are identified. A researcher should select one of the methods according to the research aim and depending on complexity of the molecular system to be studied.

## Introduction

Computations together with X-ray, NMR, and electron microscopy have been used to study the tertiary structure of biologically important proteins and to develop drugs (Kyogoku et al. 2003). Haruki Nakamura and his group have contributed to development of computational approaches and the PDBj database (https://pdbj.org/). As known, PDB is the starting point to study a single biomolecular system and structural genomics, and those studies contribute to development of drug-discovery technologies.

The human genome includes 23,000 coding genes (International Human Genome Sequencing Consortium 2001; Venter et al. 2001). Data-driven deep learning models based on the Protein Data Bank, such as Alpha fold (Jumper et al. 2021; Mosalaganti et al. 2022), succeeded to predict precise 3D protein structures from the amino-acid sequences. Recent 76% of human-protein tertiary structures was predicted (Porta-Pardo et al. 2022). The mouse genome project elucidated the time-dependent RNA expression in each organ from embryo, ES cell, and mature mouse. The genes and other sequence data were annotated in FANTOM activities (Kawai et al. 2001; Abugessaisa et al. 2021). The ENCODE project showed the gene expression in each organ of human, and the time-dependent and organ-dependent RNA expression data were published as human cell atlas, brain atlas etc. (Regev et al. 2017; Kita et al. 2021). These approaches have indicated that transcription factors are coded in about 2000 genes (10% of genes) and that 1000 promotors exist on our genome. The transcription factors bind to other proteins and form functional transcription-factor complexes. Then, these complexes bind selectively to the promotors, and finally this selective binding controls pathways, which consist of functionally related proteins (Khambata-Ford et al. 2003; Babu et al. 2004). The KEGG pathway database includes about 500 pathways, and response of RNA expression patterns against 1000 chemicals were archived in the Broad Institute as a connectivity map (Kanehisa et al. 2021; Lamb et al. 2006; Musa et al. 2018). These progresses in research give new definitions of diseases, healthy, and ageing states of life. The combination of data-driven protein-complex modelling and genome-wide association study (GWAS) elucidates the structures and functions of organelles, nuclear pore, transcription factors, and membrane systems (Uffelmann et al. 2021; Mosalaganti et al. 2022).

**Fig. 1:**
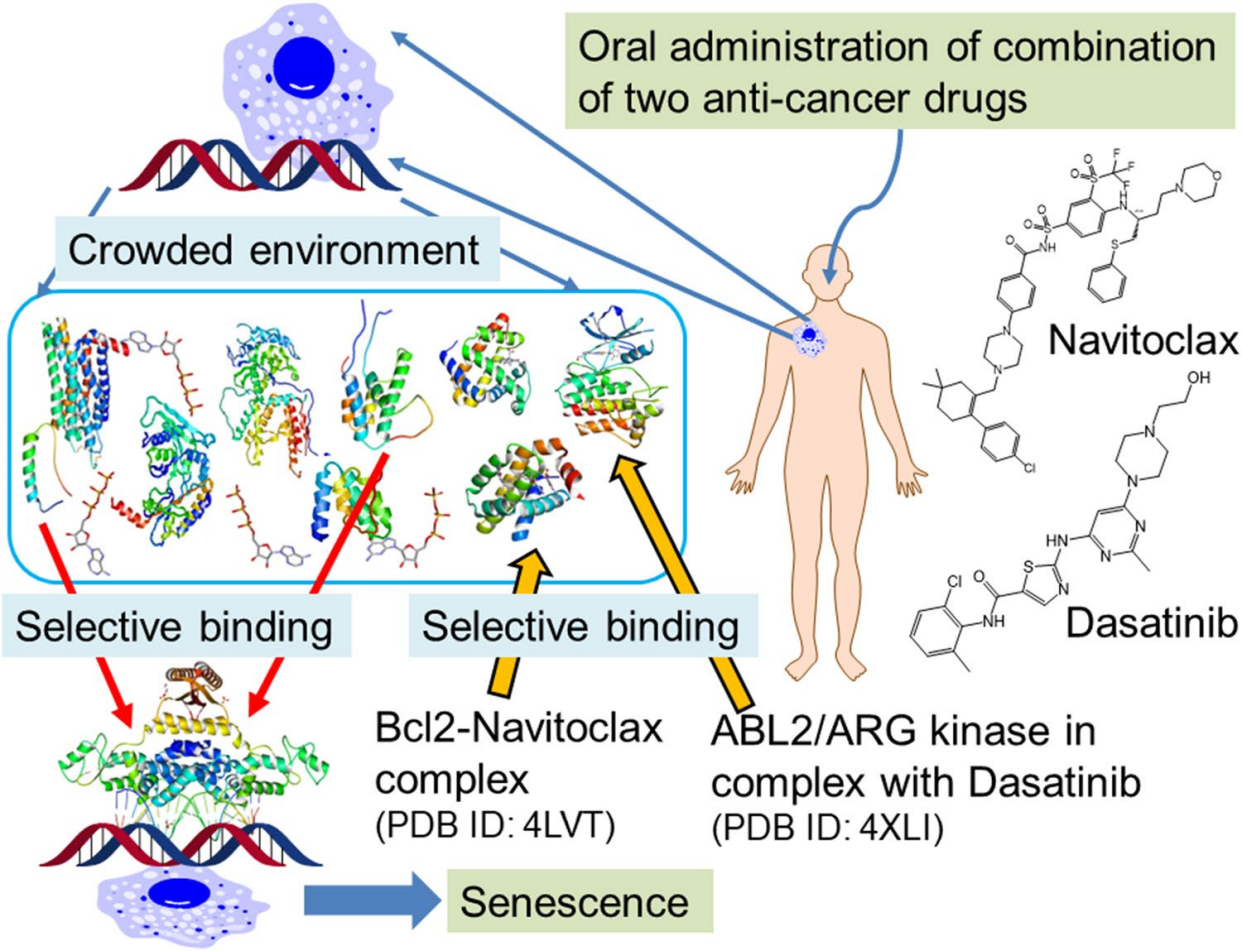
Schematic representation of systems biology, medication by drug molecules, and difficulty. Genome, transcriptome, proteome, pathway analysis, and atomic-level molecular simulation enable us to find and analyze new understandings of life and new medications

The inter-disciplinary studies reveal multiple pathway control by a combination of approved medicines. One of the successes from the inter-disciplinary studies is “chimeric antigen receptor T cell” (CAR-T cell) therapies. The genetic engineering has enabled designing artificial antibodies targeting specific antigens and these artificial genes introduced in the T cells isolated from the patient’s blood. These personalized medicines have succeeded mainly in cancer treatments. Although CAR-T cell therapies are always facing a risk of un-controlled proliferation of the CAR-T cells, some studies suggested how to control CAR-T by high-selective kinase inhibitors (Mestermann et al. 2019). Since the aging and healthy states are clearly distinguished by transcriptome and pathway analysis, some rational anti-senescence therapies have been proposed by using a pair of high-selective kinase inhibitor and Bcl-xL inhibitors (Fig. 1) (Campisi et al. 2019; Kirkland and Tchkonia 2020; Gasek et al. 2021; Shafqat et al. 2022).

The novel therapies mentioned above suggest that the state-of-the-art technology can be developed based on atomic-level interactions between the high-selective drug molecules and target proteins in solvent. This review, therefore, focusses on the computations to study the molecular interactions. How can small or medium-sized drugs and proteins bind to their target molecules selectively? The previous studies showed that each cell expresses only several thousand genes and that the produced proteins are localized in organelles (i.e., nucleus, mitochondria, endoplasmic reticulum, etc.) divided by membranes in a cell where innumerable molecules are crowded (Delarue et al. 2018; Mourão et al. 2014). As described above, these proteins bind selectively to their binding partner and form functional complexes.

The protein surfaces are mainly hydrophilic to avoid aggregation. Recently, “cryptic site” was proposed as a case of the selective binding mechanisms (Cimermancic et al. 2016; Beglov et al. 2018; Vajda et al. 2018). The cryptic site is hidden in the apo form and opened in the holo form, which is an example of polymorphism. This type of molecular recognition mechanism is understood by combination of database analysis and molecular simulations as will discussed later. The molecular simulation becomes more important when we study a binding mechanism between an intrinsically disordered protein (or domain) and its binding partner. Because a conformational motion of the intrinsically disordered protein is considerably large and complicated, a more efficient sampling method (enhanced sampling) is required, as discussed later.

Many organs (mainly brain) secrete chemicals and peptides for inter-organ cross talks. The secretions of molecules (e.g., adrenalin, histamine, insulin, endothelin) mainly work for signal transduction as stress response. In our body, most of these molecules are generated from stocked materials like amino acids, lipids, and nucleic acid. For instance, adrenalin, histamine, and dopamine are respectively generated from Phe, His, and Trp, and their chemical formulas are similar mutually. Nonetheless, the secreted chemicals are selectively recognized by their receptors (Joedicke et al. 2018). To uncover such a high selective binding mechanism, an atom-based approach is mandatory.

As mentioned above, the pathway is controlled by the protein–ligand complex formation, and then a molecule, which binds to a pathway-relating protein, can be a drug candidate. In this review, we focused on various the computational approaches from in silico (virtual) screening to enhanced sampling (generalized ensemble) to elucidate the molecular-recognition mechanism. Before that, however, we present in the next chapter a simple and fundamental framework to consider the most thermodynamically stable complex structure and semi-stable complex structures.

## Stable states

**Fig. 2:**
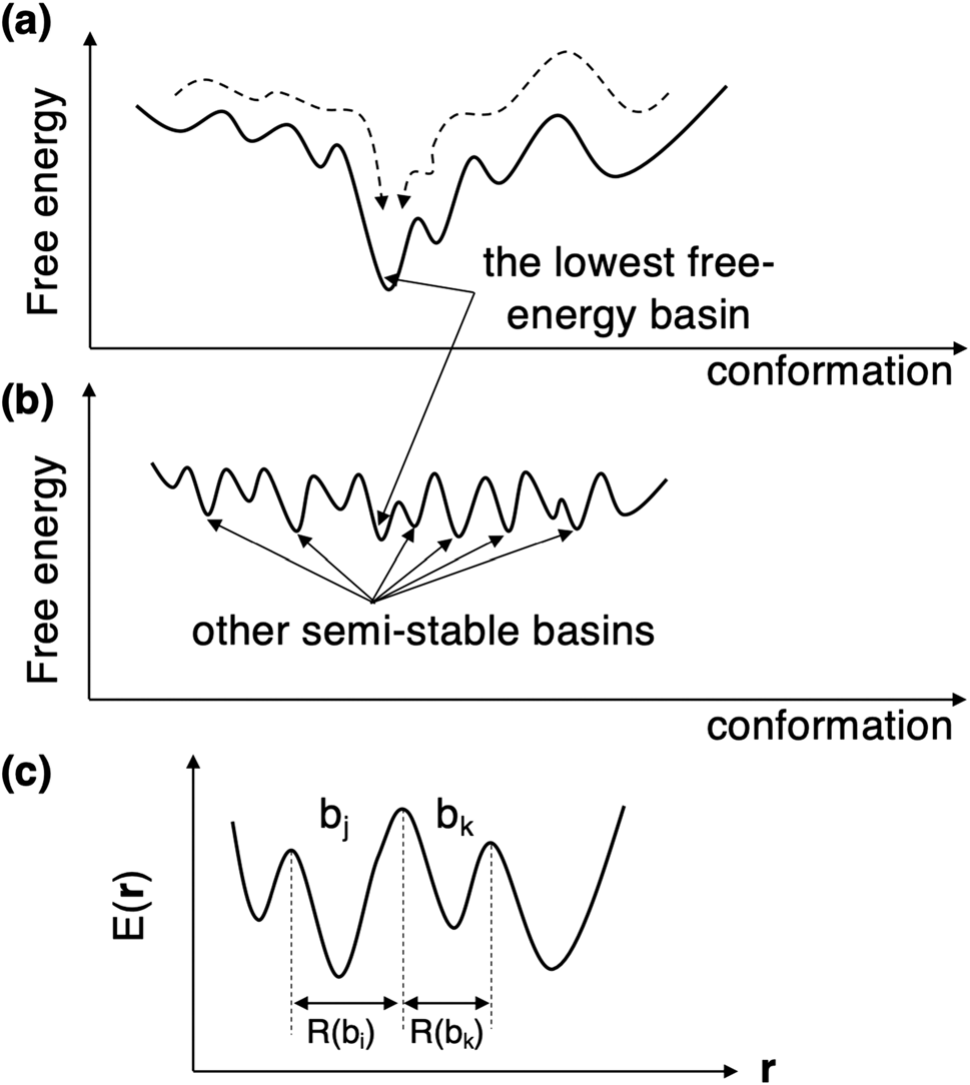
**a** Scheme of free-energy landscape. X-axis represents molecular conformation one-dimensionally, although it is high dimensional originally. Y-axis represents free energy (PMF) of conformation at physiological temperature. The lowest free energy is remarkably lower than the others. Broken lines show complex formation process. **b** System with multiple complex basins, whose free energies are similar mutually. The conformation fluctuates among the basins. **c** Potential energy surface $E({\varvec{r}})$, where ${\varvec{r}}$ is position of the system. Two basins ${b}_{j}$ and ${b}_{k}$ are mentioned in text, whose territories are $R({b}_{i})$ and $R({b}_{k})$, respectively

Before explaining the complex-structure prediction methods actually, we mention the complex formation fundamentally. Suppose that ligand and receptor are distant to each other in solution at a physiological temperature. Molecular binding is a process where the ligand approaches the ligand-binding site of the receptor and eventually the native complex is formed. In a conformational space, the binding is a process where the system’s conformation moves from a high free-energy region to a low free-energy one and finally the conformation falls in the lowest free-energy basin (native complex basin) (Fig. 2a). Contrarily, if the lowest free energy is marginally lower than the others, the system may exhibit a fuzzy complex state (Fig. 2b) (Tompa and Fuxreiter 2008), and consequently a single complex conformation is not determined experimentally because the conformation is fluctuating among multiple conformations. In this case, the aim of the computation is to find these multiple basins.

A free-energy landscape shows distribution of low free-energy basins (Fig. 2a, b). The system’s conformation ${\varvec{r}}$ is originally a multi-dimensional quantity expressed as: ${\varvec{r}}=[{x}_{1},{y}_{1},{z}_{1},\cdots ,{x}_{N},{y}_{N},{z}_{N}]$ where $[{x}_{i},{y}_{i},{z}_{i}]$ is the Cartesian coordinates of the $i$ th atom and $N$ is respectively the number of constituent atoms of the system (biological molecules, solvent molecules and other atoms in the system). Denoting the potential energy of a conformation (microscopic state) as $E({\varvec{r}})$, the statistical weight (thermodynamic weight) at thermal equilibrium assigned to ${\varvec{r}}$ at a temperature $T$ is given formally by

1 $$\rho \left({\varvec{r}}\right)\propto \mathrm{exp}\left[-\frac{E\left({\varvec{r}}\right)}{{R}_{gas}T}\right]$$

where ${R}_{gas}$ is the gas constant (the energy unit is kcal/mol). We omit a kinetic energy in Eq. 1 to make explanation simple. The normalization factor (partition function) is also omitted because $N$, system’s volume and $T$ are constant here. A fractional free energy ${G}_{{b}_{j}}$ assigned to a basin $j$ (denoted as ${b}_{j}$ in Fig. 2c) is defined by

2 $${G}_{{b}_{j}}=-{R}_{gas}Tln\left[{\int }_{R({b}_{j})}\rho \left({\varvec{r}}\right)d{\varvec{r}}\right]$$

The multi-dimensional integral is taken in a region $R({b}_{j})$ (i.e., territory of ${b}_{j}$), which is occupied by microscopic states belonging to ${b}_{j}$. Figure 2c is presented so that ${b}_{j}$ is more stable than ${b}_{k}$ at equilibrium: ${G}_{{b}_{j}}<{G}_{{b}_{k}}$.

Equation 2 is a formal expression to assess the stability of each basin. However, this multidimensional integral is not achievable for many biological systems because the high-dimensional space is fractioned into basins of complicated shapes. Instead, a ratio ${G}_{{b}_{j}}/{G}_{{b}_{k}}$ is computable numerically by an enhanced sampling simulation, whereas each of the fractional free energies ${G}_{{b}_{j}}$ and ${G}_{{b}_{k}}$ is not computable. Although rigorous determination of territory $R({b}_{j})$ is difficult, $\mathrm{exp}[-\frac{E\left({\varvec{r}}\right)}{{R}_{gas}T}]$ around an inter-basin boundary is small. Therefore, an error caused by uncertainty of $R({b}_{j})$ may be negligible.

It is helpful to convert the position ${\varvec{r}}$ defined in the Cartesian-coordinate space to a low-dimensional position ${\varvec{q}}$, refer to as “reaction coordinate”: ${\varvec{q}}=[{q}_{1},{q}_{2},\dots ,{q}_{n}]$, where $n$ is dimensionality of the reaction-coordinate space ($n<N$). Note that the function form of ${\varvec{q}}={\varvec{q}}({\varvec{r}})$ is known for the coordinate conversion. Accordingly, the weight $\rho \left({\varvec{r}}\right)$ is converted to $P\left({\varvec{q}}\right)$ as:

3 $$P\left({\varvec{q}}\right)=\int \rho \left({{\varvec{r}}}^{\boldsymbol{^{\prime}}}\right)\delta ({\varvec{q}}({\varvec{r}}),{\varvec{q}}({{\varvec{r}}}^{\boldsymbol{^{\prime}}})){d{\varvec{r}}}^{\boldsymbol{^{\prime}}}$$

where $\delta \left({\varvec{q}}({\varvec{r}}),{\varvec{q}}({{\varvec{r}}}^{\boldsymbol{^{\prime}}})\right)$ is a delta function that is non-zero only when ${{\varvec{r}}}^{\boldsymbol{^{\prime}}}$ is involved in a range ${\varvec{q}}-d{\varvec{q}}\le {\varvec{q}}({{\varvec{r}}}^{\boldsymbol{^{\prime}}})\le \boldsymbol{ }{\varvec{q}}+d{\varvec{q}}$ set in the reaction-coordinate space: ${\int }_{-\infty }^{\infty }\delta ({\varvec{q}}({\varvec{r}});{\varvec{q}}({{\varvec{r}}}^{\boldsymbol{^{\prime}}})){d{\varvec{r}}}^{\boldsymbol{^{\prime}}}=1$. In a real sampling, the number of sampled conformations is finite. Then, $\delta ({\varvec{r}}-{{\varvec{r}}}^{\boldsymbol{^{\prime}}})$ is replaced by a function $D\left({\varvec{q}}({\varvec{r}});{\varvec{q}}({{\varvec{r}}}^{\boldsymbol{^{\prime}}})\right)$ in Eq. 3: $D\left({\varvec{q}}({\varvec{r}});{\varvec{q}}({{\varvec{r}}}^{\boldsymbol{^{\prime}}})\right)=v$ in a range of ${\varvec{q}}-\Delta {\varvec{q}}\le {\varvec{q}}({{\varvec{r}}}^{\boldsymbol{^{\prime}}})\le \boldsymbol{ }{\varvec{q}}+\Delta {\varvec{q}}$ and $D\left({\varvec{q}}({\varvec{r}});{\varvec{q}}({{\varvec{r}}}^{\boldsymbol{^{\prime}}})\right)=0$ outside the range with condition of ${\int }_{-\infty }^{\infty }D({\varvec{q}}({\varvec{r}});{\varvec{q}}({{\varvec{r}}}^{\boldsymbol{^{\prime}}})){d{\varvec{r}}}^{\boldsymbol{^{\prime}}}=1$. Then, $P\left({\varvec{q}}\right)=\sum_{i}{w}_{i}D({\varvec{q}}({\varvec{r}});{\varvec{q}}({{{\varvec{r}}}_{i}}^{\boldsymbol{^{\prime}}}))$, where ${w}_{i}$ is a statistical weight assigned to the $i$ th snapshot determined from the sampling.

A force ${\varvec{F}}({\varvec{q}})$ acting on the system at ${\varvec{q}}$ in the reaction-coordinate space is expressed formally as:

4 $${\varvec{F}}\left({\varvec{q}}\right)=\left[{F}_{1}({\varvec{q}}), {F}_{2}({\varvec{q}}),\dots ,{F}_{n}({\varvec{q}})\right]$$

where the $i$ th element ${F}_{i}$ is the force acting on the system at ${\varvec{q}}$ parallel to the ${q}_{i}$ axis and defined as:

5 $${F}_{i}({\varvec{q}})={{\varvec{e}}}_{i}\bullet \left[\int \delta ({\varvec{r}}-{{\varvec{r}}}^{\boldsymbol{^{\prime}}}){\varvec{f}}({{\varvec{r}}}^{\boldsymbol{^{\prime}}})\rho \left({{\varvec{r}}}^{\boldsymbol{^{\prime}}}\right){d{\varvec{r}}}^{\boldsymbol{^{\prime}}}\right]$$

where ${{\varvec{e}}}_{i}$ is the unit vector parallel to the ${q}_{i}$ axis, and ${\varvec{f}}({\varvec{r}})$ is the force acting on the system at ${\varvec{r}}$ in the Cartesian space: ${\varvec{f}}\left({\varvec{r}}\right)=-grad\left[E\left({\varvec{r}}\right)\right]$, where derivatives are calculated with respect to the Cartesian coordinates ${\varvec{r}}$. Equation 5 indicates that ${\varvec{F}}\left({\varvec{q}}\right)$ is related to the thermal average of force ${\varvec{f}}({\varvec{r}})$ at ${\varvec{q}}$ because the thermodynamic weight $\rho \left({\varvec{r}}\right)$ is used for averaging ${\varvec{f}}({\varvec{r}})$. Therefore, ${\varvec{F}}\left({\varvec{q}}\right)$ is called a “mean force.” Then a potential function computed from a line integral of ${\varvec{F}}\left({\varvec{q}}\right)$ is called “potential of mean force” (PMF) (Tuckerman 2010). However, instead of executing the line integral, PMF is computable directly from $P\left({\varvec{q}}\right)$ as:

6 $$PMF({\varvec{q}})=-{R}_{gas}Tln[P\left({\varvec{q}}\right)]$$

The fractional free energy ${G}_{{b}_{j}}$ is computed by integrating $P\left({\varvec{q}}\right)$ in its territory $R({b}_{j})$:

7 $$\begin{array}{c}{G}_{{b}_{j}}=-{R}_{gas}Tln\left[{\int }_{R({b}_{j})}\mathrm{exp}[-\frac{PMF({\varvec{q}})}{RT}]d{\varvec{q}}\right]\\ ={\int }_{R\left({b}_{j}\right)}P\left({\varvec{q}}\right)d{\varvec{q}}\\ = {\sum }_{i}^{{n}_{j}}{w}_{i}^{{b}_{j}},\end{array}$$

where ${w}_{i}^{{b}_{j}}$ is a statistical weight assigned to the $i$ th snapshot in ${b}_{j}$, and ${n}_{j}$ is the number of snapshots in ${b}_{j}$. Although it is difficult to calculate ${w}_{i}^{{b}_{j}}$ by a conventional MD simulation in a wide conformational space, a generalized ensemble method provides ${w}_{i}^{{b}_{j}}$ naturally.

Here, we define the word “free-energy landscape” clearly. Originally, the word “free energy” is used to express an entire statistical property of the system: $G=-{R}_{gas}Tln[Z]$. The term $Z$ is the, so-called, partition function defined as $Z={\int }^{\mathrm{entire}}\rho \left({\varvec{r}}\right)d{\varvec{r}}={\int }^{\mathrm{entire}}P\left({\varvec{q}}\right)d{\varvec{q}}$, where the integral is taken over the entire conformational space. On the other hand, the word “free-energy landscape” is usually used to show the spatial patterns of the probability $P({\varvec{q}})$ or $PMF({\varvec{q}})$ in the reaction-coordinate space. Therefore, the free-energy landscape may be called “PMF landscape” or “probability landscape.” We note that the formulations of the free energy and PMF have a similarity: When $P\left({\varvec{q}}\right)$ in Eq. 6 is replaced by $Z$, $PMF$ becomes $G$.

Now we outline the computational methods and their limits of applications. If the intra-molecular deformation in each of receptor and ligand is small upon binding, a simple in silico docking is useful: A chemically stable ligand structure and the receptor’s apo form are combined as building brocks to generate various complex poses. As explained later, the ligand conformational varieties caused by rotatable-bond rotations are considered in the in silico docking. Then, the plausibility of each pose is assessed by a physical interaction energy or an empirically introduced scoring function, which is given later. Because this procedure can be done very quickly, many ligands can be tested by repeating this procedure (high-throughput screening). Details are explained later.

If the receptor undergoes a large intra-molecular deformation during the complex formation, preparation of various receptor’s conformations (ensemble) in advance is useful: The docking procedure is performed between the ligand and many conformations in the receptor’s ensemble. This procedure is called “ensemble docking” (Carlson et al. 1999; Amaro et al. 2018; Falcon et al. 2019). If this procedure works well, the complex formation accords probably to “conformation selection” (Bosshard 2001; James and Tawfik 2003; Yamane et al. 2010). The ensemble is generated from the receptor’s apo form using conventional molecular dynamics (MD), Monte-Carlo (MC) sampling, or enhanced sampling (generalized ensemble method).

If both the receptor and ligand undergo large conformational deformations during the complex formation, preparation of a ligand’s conformational ensemble as well as the receptor’s conformational ensemble may be useful. However, this procedure might be inefficient when the generated ensembles do not contain ligand and receptor conformations appropriate for constructing the lowest free-energy form (the native complex structure). This suggests that the complex formation accords with the “induced fit” (Monod et al. 1965; Spolar and Record 1994). Furthermore, a difficulty appears when conformational fluctuations (i.e., entropy) and a solvent effect contribute to the complex stability.

An extreme case is found in an intrinsic disordered segment binding to its binding partner (Wright and Dyson 1999). This segment is disordered in the unbound state and may fold in a tertiary structure when binding to the partner (coupled folding and binding) (Dyson and Wright 2005; Sugase et al. 2007). To predict the complex structure, all molecules should be involved in a single system using a completely flexible model. Therefore, a powerful sampling method, a generalized ensemble method (an enhanced sampling method), is required.

It is fundamentally interesting to distinguish the population selection and the induced fit in the complex formation (Hammes et al. 2009). Many works argued the population-selection vs induced-fit problem (Okazaki and Takada 2008; Hammes et al. 2009; Silva et al. 2011; Bucher et al. 2011; Vogt and Cera 2012; Nussinov et al. 2014; Ravasio et al. 2019; Vauquelin and Maes 2021). A generalized-ensemble study by Nakamura and his coworkers (Higo et al. 2011) reproduced a coupled folding and binding phenomena, which is expressed by an intrinsically disordered segment NRAF/REST binding to the paired amphipathic helix (PAH) domain of mSin3B (Nomura et al. 2005). The study concluded that the population selection and the induced fit works together in a coupled manner. It is natural to consider that the binding mechanism depends on the system because of the variety of the biological system.

## Receptor-ligand docking and in silico screening

Receptor–ligand docking software that predicts the receptor–ligand complex structures and the binding free energies $\Delta G$, has been a key technology of the in silico (virtual) drug screenings and the rational drug designs from 1990, and still now a number of reports has been published on the receptor–ligand docking programs and the combinations of them (Pagadala et al. 2017; Salmaso and Moro 2018; Amaro et al. 2018; Bender et al. 2021; Pinzi and Rastelli 2019). Haruki Nakamura and his coworkers are developers of docking software (sievgene/myPresto) and a basic method for docking study (Fukunishi et al. 2005). Part of his work is now available as “myPresto program suite” (https://www.mypresto5.jp/en/) where about 20 programs can be downloaded under a LGPL v2 license. A member of myPresto software developers is allowed to use them under FreeBSD license.

Before starting the docking procedure, the ligand-binding site must be indicated (this identification is discussed later). In general, a docking method consist of two or three steps. The first step is the ligand-allocation scheme on the receptor surface around the indicated ligand-binding site and gives many receptor–ligand complex structure candidates (“docking poses”). The second step is an evaluation of the docking poses by applying a scoring function, which estimates roughly the $\Delta G$ values of given docking poses and selects some probable or stable docking poses. The third step is the re-scoring of the selected poses by using a more precise scoring function than the rough scoring function used above. The final docking poses correspond to $\Delta G$ values.

Usually, the scoring function is classified into three types (Li et al. 2019): physico-chemical, knowledge-based, and empirical scoring functions. We focus on the docking methods based on the physico-chemical scoring function because this scoring function can incorporate readily new elements, such as boron (Soriano-Ursúa et al. 2014) and silicon (Franz and Wilson 2013). The popular docking software based on the physico-chemical scoring is Dock (Kuntz et al. 1982), AutoDock (Goodsel et al. 1996), Glide (Friesner et al. 2004; Halgren et al. 2004) etc. The knowledge-based scoring function is calculated from a pairing distribution function between two atom-groups recorded in a database. The popular docking software based on the knowledge-based scoring function are GOLD (Jones et al. 1997; Verdonk et al. 2003). The empirical one is from parameter tiffing in a function to reproduce the experimental $\Delta G$ (Pereira et al. 2016; Ragoza et al. 2017). The popular docking software based on the empirical base is GOLD chemscore, FlexX (Rarey et al. 1996), PRO-LEAD (Baxter et al. 1998), rDock(Ruiz-Carmona et al. 2014) etc.

## Binding-free energy estimation is the core technology of docking software

Receptor–ligand binding free energy is one of the major factors determining the activities of drug molecules, signal transductions, and many other physiological phenomena. Both the molecular simulation and experimental approaches can give the $\Delta G$ values. Suppose that one receptor has only one ligand-binding site (or region) and that one receptor molecule can bind only one ligand molecule to form one receptor-ligand complex. Let the ligand-binding region (${R}_{B}$) be clearly distinguishable from the other region (${R}_{U}$) in the conformational space. Equations 3 and 7 gives $\Delta G$ $(={G}_{{R}_{B}}-{G}_{{R}_{U}})$ as follows,

8 $$\Delta G=-{R}_{gas}Tln\left[\frac{{\int }_{{R}_{B}}P\left({\varvec{q}}\right)d{\varvec{q}}}{{\int }_{{R}_{U}}P\left({\varvec{q}}\right)d{\varvec{q}}}\right]=-{R}_{gas}Tln\left[\frac{{P}_{B}}{{P}_{U}}\right]$$

where ${P}_{B}$ and ${P}_{U}$ are the probabilities of the bound (${R}_{B}$) and unbound (${R}_{U}$) states, respectively: ${P}_{B}=\left[{\int }_{{R}_{B}}\rho \left({\varvec{r}}\right)d{\varvec{r}}\right]$ and ${P}_{U}=\left[{\int }_{{R}_{U}}\rho \left({\varvec{r}}\right)d{\varvec{r}}\right]$ using Eq. 3.

On the other hand, the popular experimental methods for $\Delta G$ evaluation are the isothermal titration calorimetry (ITC) and the surface plasmon resonance (SPR) experiments that give the binding constant ${K}_{a}$ (Rich and Myszka. 2007; Wiseman et al. 1989). When the system is in the equilibrium state under the standard condition (298 K and 1 atm), the dissociation constant ${K}_{D}(=1/{K}_{a})$ gives the standard molar Gibbs free energy change of binding $\Delta {G}^{0}$ as follows,

9 $$\Delta {G}^{0}={R}_{gas}Tln\left[\frac{{K}_{D}}{{C}^{0}}\right]$$

where ${C}^{0}$ is a reference concentration of 1 mol/L (Gilson et al. 1997; Deng and Roux 2009).

Since the $\Delta G$ value depends on the experimental conditions (temperature, pressure, and the other experimental conditions), $\Delta {G}^{0}$ is useful for comparing the stability of complexes among multiple receptors and ligands measured from different experiments. Therefore, $\Delta {G}^{0}$ is adopted in the scoring functions.

Besides the binding-constant observation experiments, there have been many experimental methods, which provide the binding affinity of ligand. Namely, the half maximal inhibitory concentration (${IC}_{50}$), percent inhibition, inhibition constant (${K}_{i}$) etc. These quantities could be somehow translated to the binding free energy differences by using the Cheng-Prusoff equation (Yung-Chi and Prusoff 1973) and the other equations. These affinity data have been useful for developing the scoring functions.

As mentioned, the accurate calculation of $\Delta G$ is still a very time-consuming and expensive task. On the other hand, in silico screening is usually applied to many compounds. Preparation of large ligand library and usage of many computation nodes are effective to increase the efficiency of the in silico screening (Gentile et al. 2022; Gorgulla et al. 2020; Lyu et al. 2019). Therefore, approximation of $\Delta G$ with maintaining a certain accuracy is crucially important for docking software.

## Receptor–ligand docking as supporting tool for X-ray crystallography

In 1983, Kuntz group published the first docking program DOCK for assisting the X-ray crystallographic coordinates of small molecules in the protein–ligand complex (Kuntz et al. 1982). The docking procedure of DOCK was following four steps. (1) DOCK puts various size of spheres that represent the ligand on the receptor surface to search the ligand-binding position with avoiding atomic conflictions. (2) The second step is a trial-and-error ligand-docking cycle. DOCK locates the ligand molecule in various conformations on the predicted ligand-binding position indicated by Step 1. (3) DOCK evaluates the stability of each conformation by applying a binding-enthalpy function. (4) Finally, DOCK selects the candidate most-stable receptor-ligand conformation. DOCK estimates the binding enthalpy ($\Delta E$) by Eq. 10 instead of Eq. 11 which is the classical force field for an MD simulation.

10 $$\Delta E=\sum\nolimits_{i\in rec}\sum\nolimits_{j\in rec}4{\varepsilon }_{ij}\left\{{\left(\frac{{\sigma }_{ij}}{{r}_{ij}}\right)}^{9}-{\left(\frac{{\sigma }_{ij}}{{r}_{ij}}\right)}^{6}\right\}+332.0\sum\nolimits_{i\in rec}\sum\nolimits_{j\in rec}\frac{{q}_{i}{q}_{j}}{4{{r}_{ij}}^{2}}$$

or

11 $$\Delta E=\sum\nolimits_{i\in rec}\sum\nolimits_{j\in rec}4{\varepsilon }_{ij}\left\{{\left(\frac{{\sigma }_{ij}}{{r}_{ij}}\right)}^{12}-{\left(\frac{{\sigma }_{ij}}{{r}_{ij}}\right)}^{6}\right\}+332.0\sum\nolimits_{i\in rec}\sum\nolimits_{j\in rec}\frac{{q}_{i}{q}_{j}}{{r}_{ij}}$$

where subscripts $i$ and $j$ designate the $i$ th atom of receptor and the $j$ th atom of ligand, respectively. The parameters ${r}_{ij}$, ${\sigma }_{ij}$, and ${\varepsilon }_{ij}$ are the inter-atomic distance (Å), van der Waals (vdW) radius (Å), and a coefficient for the vdW interaction assigned to the atom pair of $i$ and $j$. The parameters ${q}_{i}$ and ${q}_{j}$ are atomic partial charges (atomic unit) assigned to atoms $i$ and $j$, respectively. The number “332.0” is to set the energy in kcal/mol unit. The first and second terms of Eq. 10 correspond respectively to the soft-core vdW interaction and the Coulomb interaction in implicit-water solvent. In general, a receptor–ligand complex with a strong affinity shows good interface complementarity. A slight coordinate error of the ligand causes atomic conflicts, which result in a strong repulsion and a large error in the docking score. Therefore, most receptor–ligand docking programs have adopted the soft-core vdW potential.

In the Coulomb interaction term, $4{r}_{ij}$ represents an effective dielectric constant ${\varepsilon }_{eff}$. Assuming that ${\varepsilon }_{eff}$ depends on the distance ${R}_{p}$ from the protein surface to the ligand, the simplest form is ${\varepsilon }_{eff}=4{R}_{p}$ (Mallik et al. 2002). In Eq. 10, ${R}_{p}$ is approximated by ${r}_{ij}$.

Because the initial version of DOCK was designed for crystal-structure analysis, this version did not involve an entropy term. DOCK has been modified frequently last few decades, and the current ligand-allocation scheme and the scoring function are different from those of the initial version.

## Toward receptor-ligand docking in implicit aqueous solvent

Present docking software is designed to the receptor–ligand docking in implicit water solvent at the room temperature. Equation 12 is one of the AutoDock scoring functions, and many other scoring functions are similar to this function more or less (Goodsel et al. 1996). Note that $\Delta G$ in Eq. 12 is regarded as an approximation of the free energy change caused by the ligand-receptor complex formation. The enthalpy part of $\Delta G$ is expressed by the first three terms: the receptor–ligand vdW, hydrogen-bonding, and Coulomb interactions. The entropy part of $\Delta G$ consists of the fourth and last terms. The fourth term represents the entropy loss of ligand molecule in binding: the ligand can have multiple conformations in bulk water (unbound state), although it is fixed to a single conformation in the binding site. The last term represents the receptor–ligand hydrophobic interaction in water.

12 $$\begin{array}{c}\Delta G={f}_{vdW}\sum\limits_{i,j}\left(\frac{{A}_{ij}}{{r}_{ij}^{12}}+\frac{{B}_{ij}}{{r}_{ij}^{6}}\right)+{f}_{hbond} \left\{\sum\limits_{i,j}\left(\frac{{C}_{ij}}{{r}_{ij}^{12}}+\frac{{D}_{ij}}{{r}_{ij}^{10}}\right)+{E}_{hbond}\right\}\\ +{f}_{elec}\sum_{i,j}\frac{{q}_{i}{q}_{j}}{\varepsilon ({r}_{ij}){r}_{ij}}+\Delta {G}_{tor}{N}_{tor}+{f}_{sol}\sum_{i,j}\left({S}_{i}{V}_{j}+{S}_{j}{V}_{i}\right)\mathrm{exp}\left[-\frac{{r}_{ij}^{2}}{2{\sigma }^{2}}\right],\end{array}$$

where ${f}_{vdW}$*, *${f}_{hbond}$*, *${f}_{ele}$*,* ${f}_{sol}$, and ${f}_{vdW}$ are respectively fitting coefficients for terms of vdW, hydrogen bond, Coulomb, entropy-loss, and the dehydration free energy to remove a hydration shell from the receptor–ligand interface. A classical MD force field gives the values of coefficients ${A}_{ij}$, ${B}_{ij}$, ${C}_{ij}$, and ${D}_{ij}$. ${E}_{hbond}$, ${q}_{k}$ ($k=i \mathrm{or} j$), $\varepsilon \left({r}_{ij}\right)$, $\Delta {G}_{tor}$, and ${N}_{rot}$ are respectively a correction term for a hydrogen bond, an atomic charge for atom $k$, the distance-dependent dielectric constant for atom pair $i$ and $j$, the entropy loss with respect to a rotatable bond, and the number of rotatable bonds in the ligand. ${S}_{k}$, ${V}_{k}$, and $\sigma$ are respectively an atomic-solvation parameter for atom $k$, an occupied atomic volume for atom $k$, and an average vdW radius of heavy atom except hydrogen atom. The fitting coefficients are determined to reproduce the experimental $\Delta G$ values from many receptor–compound complexes.

**Fig. 3:**
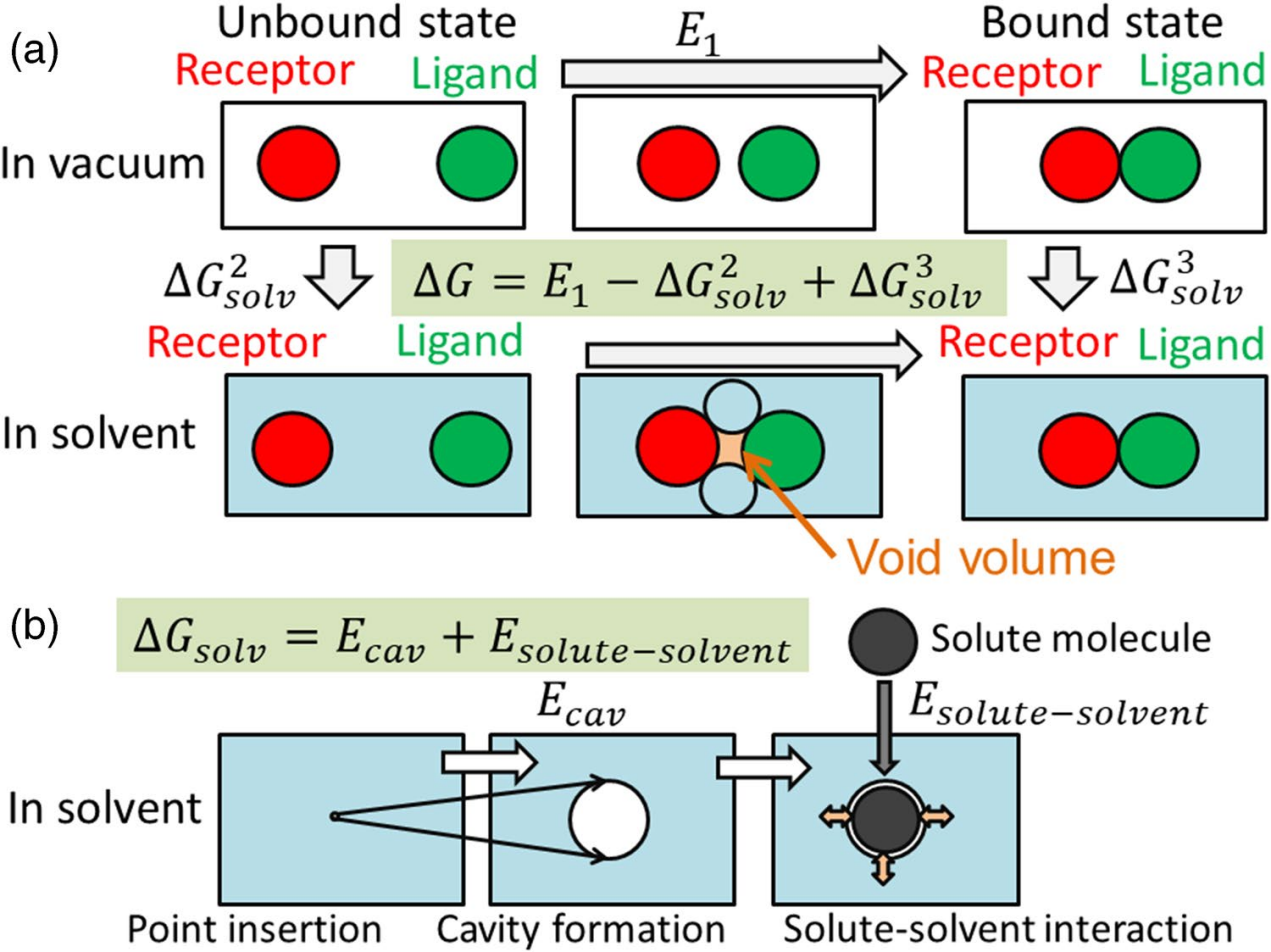
Schematic representation of molecular interaction energy calculation. **a** Thermodynamic cycle to calculate $\Delta G$. ${E}_{1}$, ${\Delta G}_{solv}^{2}$ and ${\Delta G}_{solv}^{3}$ are transfer energies and arrows represent the transfer directions. Boxes in blue represent solvent water. Red, green, and blue circles represent the receptor, ligand, and water molecules, respectively. Void volume is colored in orange between the receptor, ligand, and water molecules. **b** Schematic representation of scaled-particle theory. Point insertion, cavity formation, and solute–solvent interaction energy are shown. Small arrows in orange represent the solute–solvent interaction

The parameters in the scoring function $\Delta G$ are determined from a thermodynamic cycle, which is a well-known cycle in computational field as shown in Fig. 3a and b. Figure 3a shows that $\Delta G$ is given by the molecular interaction in vacuum (${E}_{1}$ ) and the aqueous solvation free energies ($\Delta {G}_{solv}^{2}$ and $\Delta {G}_{solv}^{3}$ ). Since classical force field gives the ${E}_{1}$ value, the unknown factor is only the solvation free energy. Figure 3b shows that the solvation process consists of the cavity formation and solute–solvent interaction processes. Broadly speaking, the works for cavity formation and the short-range solute–solvent interaction energy are approximately proportional to the surface area of the cavity. The fitting parameters of the surface area are determined to reproduce the experimental solvation free energy values of various compounds. Thus, the physico-chemical docking scoring function is, in general, a combination of surface area term and the classical force field used in the conventional molecular simulation.

## Estimation of cavity formation energy in aqueous solvent

The scaled particle theory (SPT) has been one of the basic theories for solvation energy calculation and most of the docking software adopts the SPT or variations of SPT (Pierotti 1976). The original SPT explains the solvation of one spherical particle in the solvent and the SPT was extended to estimation of solvation energy of receptor-ligand systems. Namely, the result obtained by the SPT shows that the solvation free energy is approximately proportional to the solute surface area. By replacing the radius of spherical solute by the solvent-accessible surface area, the approximation formula of solvation free energy given by SPT is extended for solvation of polyatomic molecules. Finally, the approximation formula is extended to estimation of solvation-free energy of the receptor-ligand systems.

In SPT, the solvation consists of two processes (Fig. 3b). The first process is a vacuum cavity formation for insertion of solute in the solvent, and the second process is calculation of a solute–solvent interaction when the solute exists in the cavity. The cavity formation energy ${E}_{cav}$ is approximated by a polynomial as follows.

13 $${E}_{cav}={c}_{0}+{c}_{1}R+{c}_{2}{R}^{2}+{c}_{3}{R}^{3}+{c}_{4}{R}^{4}+\cdots ,$$

where $R$ is the radius of the cavity, and ${c}_{k}$ ($k=\mathrm{1,2},\dots$) is a coefficient assigned to each term. This equation is an expansion of a general equation ${E}_{cav}=-{R}_{gas}Tln\left[\rho \right]$, where $\rho$ is the atomic packing factor.

Each term of Eq. 13 has its own physico-chemical significancy although we do not explain in detail: See paper by Pierotti (1976) for instance. When the solvent is water at pressure 1 atm and when it consists of a spherical-rigid water model, SPT shows that the radius of solvent molecule and the density of solvent determine the ${c}_{0}$, ${c}_{1}$, ${c}_{2}$ and ${c}_{3}$ values and the other higher order coefficients are zero, and the third term (${c}_{2}{R}^{2}$) is dominant. Then, Eq. 13 is rewritten as

14 $${E}_{cav}\approx {c}_{2}{{R}^{^{\prime}}}^{2}={c}_{surf}\times ASA ,$$

where ${c}_{surf}$ and $ASA$ are the coefficient of atomic surface tension and the solvent-accessible surface area (ASA) of the given solute, respectively. $R$ is replaced by ${R}^{^{\prime}}$, which is sum of $R$ and the vdW radius of a water molecule. While $ASA$ is a quantity difficult to be computed for a solute of general shape, Richmond provided an analytical computation method (Richmond 1984). However, the computation was still time consuming by a computer. Then, Stouten et al. proposed a simple and fast approximation method without conditional branch (Stouten et al. 1993) as follows.

15 $${E}_{cav}={f}_{sol}\sum_{i,j}\left({S}_{i}{V}_{j}+{S}_{j}{V}_{i}\right)\mathrm{exp}\left[-\frac{{r}_{ij}^{2}}{2{\sigma }^{2}}\right]$$

Note that this expression appears in the last term of Eq. 12. Now, this approximation and the variations have been widely used, e.g., AutoDock (Goodsel et al. 1996) and sievgene (Fukunishi et al. 2005).

## Solvation free energy

Major inter-molecular interactions for biomolecules are the vdW and Coulomb interactions. Ooi et al. assumed that the vdW interaction is a short-range interaction and that the major contribution of the electrostatic interaction is from the first hydration shell (Ooi et al. 1987). Then, both ${E}_{cav}$ and the solute–solvent interaction energy, ${E}_{solute-solvent}$, are proportional to ASA approximately, and the solvation free energy $\Delta {G}_{solv}$ is given simply as

16 $$\Delta {G}_{solv}={E}_{cav}+{E}_{solute-solvent}\approx c\times ASA ,$$

where $c$ is, so-called, an atomic solvation parameter. Dividing $ASA$ into contribution from individual atoms, Eq. 16 is transformed as

17 $$\Delta {G}_{solv}\approx {\sum }_{i=1}^{N}{c}_{i}\times {ASA}_{i} ,$$

where ${c}_{i}$ and ${ASA}_{i}$ are the atomic solvation parameter and the ASA of the $i$ th atom, respectively, and $N$ is the number of atoms in the receptor and ligand. The parameter ${c}_{i}$ depends on the atomic partial charge and vdW parameter of each atom. Various modified ASA methods have been proposed (Kang et al. 1987).

To improve the accuracy of $\Delta {G}_{solv}$, the electrostatic energy was further considered because this energy is long range by nature. The Poisson-Boltzmann (PB) equation provides the electrostatic energy in a cell of the 3D real space consisting of multiple small volumes with different dielectric constants. On the other hand, the PB equation should be solved in a large cell to consider the long-range property of the electrostatic energy (Gilson et al. 1988). Although Nakamura et al. succeeded in solving the PB equation precisely in a small cell, the computation was still time consuming (Nakamura and Nishida 1987; Nakamura 1988). The generalized Born (GB) method is a fast approximation method for the electrostatic energy, which is designed to mimic the results from the PB equation (Hawkins et al. 1996; Onufriev et al. 2002). In the framework of the GB method, the short-range interaction is only the vdW interaction (${E}_{vdW}$), and $\Delta {G}_{solv}$ is given as

18 $$\Delta {G}_{solv}={E}_{cav}+{E}_{vdW}+{E}_{Coulomb}\approx c{\sum }_{i=1}^{N}{ASA}_{i}+{E}_{GB} ,$$

where ${E}_{Coulomb}$ is the electrostatic energy and ${E}_{GB}$ the approximated electrostatic energy from the GB equation. The atomic surface tension parameter ${c}_{i}$ in Eq. 17 is constant in Eq. 18, which is set to 10 cal/mol/Å^2^ in aqueous solvent in general. The combination of the PB equation and SPT is called a PBSA method, and Eq. 18 is called a GBSA (generalized-Born accessible-surface area) method. Currently, the GBSA method with a quantum mechanics (QM) method in the reaction field has succeeded in reproducing the solvation free energies and pKa for various solutes (Irisa et al. 1995; Cramer and Truhlar 2008).

As mentioned in Eq. 18, the atomic surface tension parameter $c$ is constant. However, the solvent structure and dynamics (entropy and enthalpy) depend on the site around protein (Suzuki et al. 1997; Assaf and Nau 2018; Salis and Ninham 2014; Nakamura et al. 1988; Lumry and Rajender 1970; Freire 2008; Kabir et al. 2003). This means that $c$ is not constant. Still now, the solvent structure on the solute–solvent interface and the change of entropy and enthalpy upon the receptor-ligand binding are unclear.

## Additional effect not included in many scoring functions: void-volume effect

The effect of a void volume to $\Delta G$ is not considered in Eq. 12. The void is defined by a volume between the Conolly and vdW surfaces in a system consists of solutes and water molecules (Fig. 3a). The contribution of the void volume to free energy is well explained by physics and the behavior of PMF by changing the void volume was computed accurately (Rashin 1989 and 1990; Fukunishi and Suzuki 1996; Gallicchio et al. 2000; Trzesniak et al. 2007). However, the estimation of the void volume contribution to $\Delta G$ is time consuming and ignored in many docking programs.

## Ligand conformation generation and force field

Before starting ligand docking, most of docking software prepares the multiple conformations of ligand with respect to rotatable bonds. Designating the number of rotatable bonds as ${N}_{rot}$ and supposing that the number of energy minima regarding the bond rotation is three, e.g., trans, gauche^+^ and gauche^−^, the number of possible stable conformations is ${3}^{{N}_{rot}}$. If the receptor-ligand complex adopts only one binding pose, the ligand selects one conformer out of the ${3}^{{N}_{rot}}$ ones and the ligand loses entropy of $ln\left[{3}^{{N}_{rot}}\right]$.

The force fields (FFs) for small compounds were estimated from X-ray diffraction data and infrared (IR) spectrums. A GF matrix (or FG) method translates the IR spectrum to the force constants of the bonds, angles, bond-angle cross terms of the molecule (Wilson 1941; Boyd 1968). Allinger et al. constructed the force fields of small compounds and developed the MM2/MMP2 and MM3 programs. MMP2 calibrates the force fields around aromatic rings by the semi-empirical QM (Allinger 1976, 1977; Allinger et al. 1994). The Quantum Chemistry Program Exchange (QCPE) distributed the programs of MM2/MMP2, ECEPP and many program-source codes to computer chemists all over the world by free (Halgren 1996a, 1996b; Boyd 2013). Halglen et al. applied the high-level ab-initio QM to many compounds and developed MMFF94 force field (Halgren 1996a, 1996b). Namely, MMFF94 force field parameters were derived from 500 molecular structures optimized at the HF/6-31G* level, 475 structures optimized at the MP2/6-31G* level, 380 MP2/6-31G* structures and 1450 structures partly derived from MP2/6-31G* geometries. The MM2/MMFF94 force fields and MM2 software have been widely used still now. Since the research purpose and force-field formula are different between the small chemical compounds and proteins, several groups have developed new force fields like the general AMBER force field (GAFF) and CHARMm, which are applicable to various biological systems including protein, RNA, DNA and drug molecules (Wang et al. 2004; Zhu et al. 2012; Kumar et al. 2020).

These force fields can be used for the conformer generation of small molecules, and conformation generators CONCORD, Corina and CONFLEX have been developed (Gasteiger et al. 1990; Osawa et al. 1989; Kotev et al. 2005). Whereas conformer generation of chain structures is easy, treatment of ring puckering (conformer generation of cyclic structure) is a difficult problem (Cremer and Pople 1975; Cremer 1990). Recent cluster analysis revealed that the number of ring conformers is considerably smaller than ${3}^{{N}_{rot}}$, that the possible torsional angles of the ring main chain are limited, and that the number of typical ring conformers increases slowly with increasing the ring member atoms (Friedrich et al. 2019; Chan et al. 2021). Such study may enable the fast ring-conformer generation including macrocycles and cyclic peptides. However, the conformers of bi-cyclic compounds are still unclear. Steric hindrance restricts the rotation around rotatable bonds (atropisomers). Use of atropisomers is an effective technique to increase the binding affinities of drug molecules, although atropisomers make the estimation of the entropy change, $\Delta S$, upon binding difficult (Toenjes and Gustafson 2018).

## Problem omitted in this chapter

In this chapter, we did not explain the solvation accompanying with quantum effects. Namely, charge transfer complexes, covalent drugs, halogen bonding, S–O interaction, metal bindings and so on. Currently, some docking programs can be applied to the covalent drugs, halogen bonding and metal binding. Chemical reactions of approved covalent drugs are mainly mild with targeting OH and SH groups of receptors and improve the drug potency and clearance (Bauer 2015).

Heavy halogen atoms (I, Br, and Cl) of ligand molecules can bind to both positively and negatively charged atoms. Each halogen atom of the molecules has one chemical bond in general and the opposite side of the halogen atom becomes positively charged, while the other part is done negatively (sigma hole effect). Thus, halogen atoms can bind to both positively and negatively charged atoms. The halogen bond is an electrostatic interaction and could be estimated in the framework of classical force fields (Harder et al. 2016).

S–O interaction is an intra-molecule $\pi$ orbital interaction between sulfur and oxygen atoms. S–O interaction is useful to fix the ligand conformer to the active coordinates for increasing the receptor-ligand binding free energy. S–O interaction is a quantum effect, and there is no classical force field that can represent this effect currently (Nagao et al. 1998).

One of the most important interactions is the metal bindings, since many enzymes contain soft metal atoms, e.g., Zn, Cu, and Fe that can change the number of valence electrons with a small energy change in the reaction centers. In general, lone-pair electrons of ligand bind to metal atoms of enzyme, and additional point charges representing the lone-pair electrons of ligand atoms can reproduce the metalloprotease–ligand complex structure. But the covalency of the metal binding makes the binding energy prediction difficult comparing to the vdW and Coulomb interactions.

## Docking scores as descriptors of the receptors and ligands: ensemble receptor-ligand docking and other applications

**Fig. 4:**
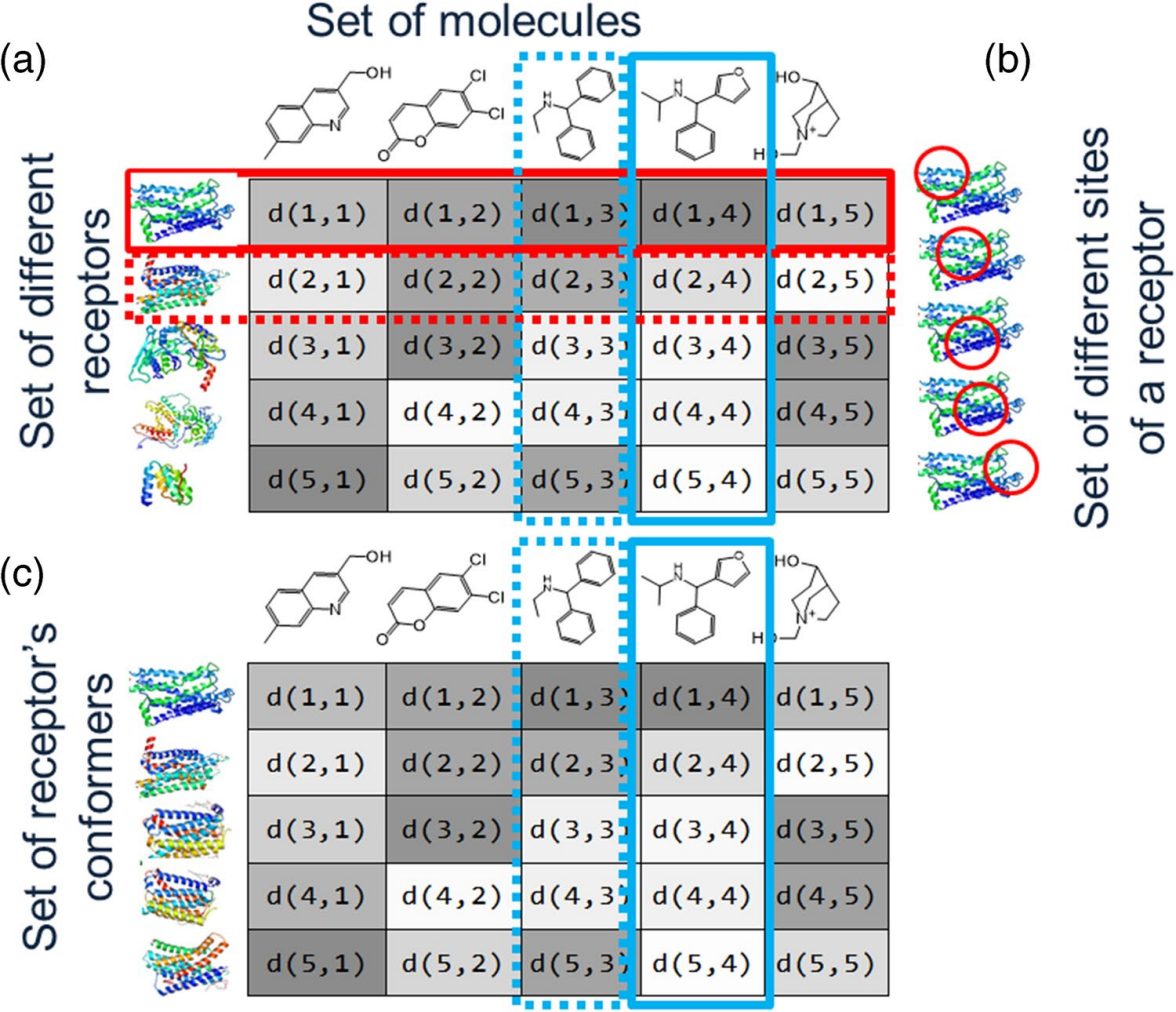
**a** Interaction table. A matrix element $d(i,j)$, is docking results (docking score and docking pose) between the $i$ th receptor and the $j$ th ligand molecule obtained from the multiple-target screening and MASC scoring methods. In this table, $i$ or $j=1,\dots ,5$. Ligand molecules in blue solid-line frame and in blue dotted-line frame are similar to each other. Receptors in red solid-line frame and in red dotted-line frame are similar to each other. In this panel, a darker tone assigned to $d(i,j)$ represent a higher score (higher affinity). **b** Red-colored circles indicate partial areas of a single receptor. Ligand docking is restricted in the red circles. **c** Interaction table for ensemble docking. Elements of this table are the docking results between conformers of a single target receptor in ensemble and a set of ligand molecules

Numerous receptor-ligand associations and dissociations support life activities. Therefore, the receptor-ligand docking results can be useful descriptors for predicting various biological phenomena. Let think about the docking results among all receptors and all compounds in our body, where each docking result is a pair of the docking score (or $\Delta G$) and the docking pose. Figure 4a is an interaction table between five receptors and five ligand molecules, and Fig. 4c is an interaction table between five conformers of a single receptor and the five ligand molecules. Assume that a matrix element $d(i,j)$ is assigned to the $i$ th receptor and the $j$ th compound in Fig. 4a. Similarly, a matrix element $d(i,j)$ is assigned to the $i$ th conformer of a single receptor and the $j$ th compound in Fig. 4c. The element $d(i,j)$ represents a pair of the docking score ${s}_{ij}$ and the docking pose. The docking pose is described in many ways: The 3D Cartesian coordinates of the receptor-ligand complex structure, vector representations that represent receptor-ligand interactions, 3D and 4D grid representations that represent the distribution of ligand’s substructures and so on (Deng et al. 2004; Fujita and Orita 2008). Here after, we call the docking results among the multiple-receptor structures and multiple-compounds summarized in Fig. 4a and b as “interaction table” and discuss some applications of the tables.

Let the first receptor in Fig. 4a be the target receptor. The red solid-line frame in Fig. 4a represents the docking results of the ligands to the target receptor. Sorting $d(1,j)$ ($j=1$ to 5) in descending order of ${s}_{1j}$ is the conventional docking screening by the score. Note that once the set of multiple docking data is described as a matrix, we can apply various mathematical matrix operations to the matrix. These operations correspond to the chemical applications of the interaction table. In the following section, we introduce some applications of the interaction table.

## Docking screening for choosing target-selective molecules based on the interaction table

Because many kinds of receptors exist in our body, drug molecules must bind specifically to its target receptor. Otherwise, the low selectivity and off-target binding may cause a side effect or adverse effect. Suppose that Molecules A and B bind to a target receptor, and that the binding energy for Molecule A is stronger than that for Molecule B. If we select the molecule with the strongest binding energy, Molecule A should be the hit compound. However, if Molecule A bind to other receptors much more strongly than to the target receptor and if Molecule B does not bind to other receptors, we should select Molecule B as the hit compound. This example suggests that the in silico screening for a target receptor needs some additional docking studies for other receptors.

The multiple target screening (MTS) method is an in silico screening method to choose the target-selective molecules (Fukunishi et al. 2006b). This method is simple. Each molecule in the compound database (DB) is docked to proteins of a protein set (each column of the interaction table: Fig. 4a). The molecules with a higher score to the target than that to the other receptors are considered as target-selective molecules.

Figure 4a exemplifies that the fourth compound by the blue solid-line frame is the candidate hit molecule for the first receptor indicated by the red solid-line frame. If the rank of the docking score between the target receptor and the ligand represents the target-selectivity to the ligands, we can select the ligand whose docking score to the target receptor is top-ranked as candidate hit molecule.

The other method is to use a deviation of docking score (MASC score) instead of the intact docking score (Vigers and Rizzi 2004). The MASC scoring method assumes that each ligand molecule has its own average docking score to a set of receptors. Target-selective molecules should show strong docking scores to the target receptor, while the same molecules should show weak docking scores to the other receptor. Thus, the target-selectivity of a ligand molecule should correspond to the *z*-score (deviation) of the docking score to the target receptor among the docking scores to many receptors. The MASC scoring method selects the highest z-score molecules as the hit compounds.

## Improvement of docking results by machine-learning approaches based on the interaction table

Similar receptors tend to bind to similar ligands: Subtypes of receptors belonging to the same protein family bind to the same or similar ligand (i.e., Kinase family, GPCR family etc.). Thus, we can expect that a weighted average of the docking scores of over multiple receptors is more reliable than that based on a single receptor structure. The weight for each receptor depends on the degree of the similarity among the receptors. Then an averaged docking score, ${S}_{i}^{a}$, between a receptor $a$ and a ligand $i$ is defined as

19 $${S}_{i}^{a}=\sum\nolimits_{b}{w}_{b}^{a}{s}_{i}^{b}$$

where ${s}_{j}^{b}$ is the docking score for complex of receptor $a$ and ligand $i$ (i.e., the score in the interaction table), and ${w}_{b}^{a}$ is a weight representing the contribution of ${s}_{j}^{b}$ to the averaged docking score ${S}_{i}^{a}$. The determination of ${w}_{a}^{b}$ can be achieved by machine-learning approaches when teaching data sets are available, which are experimental assay results (Fukunishi et al. 2006a; Fukunishi 2009).

In fact, the Nakamura group applied the docking-score QSAR method to 107 kinase assay results registered in ChEMBL database, made the prediction models for the 107 kinases based on total 20,000 ligand molecules, and reported that the average error of $\Delta G$ prediction was 0.7 kcal/mol (Fukunishi and Nakamura 2012; Fukunishi et al. 2017). Interestingly, they started the study from a general equation for ${S}_{i}^{a}$ (i.e., ${S}_{i}^{a}=f(\{{w}_{b}^{a}{s}_{j}^{b}\})$) and concluded that the simple linear equation (i.e., Eq. 19) is a good expression for ${S}_{i}^{a}$.

ChEMBL and PubChem are the most widely used public molecular-interaction repositories (Kim et al. 2021; Gaulton et al. 2017). ChEMBL31 includes the 20 million molecular interactions among 2.3 million chemicals and 15,072 proteins. PubChem does the 297 million bioactivities of 112 million compounds. Many prediction models have been developed based on these repositories (Fukunishi 2009).

## Similarity searches of molecules, receptor binding sites and ligand-based drug screening based on interaction table

The interaction table determines both the similarities among receptors and those among ligand molecules. If the structures of the $a$ th and $b$ th receptors are similar, the vector for the $a$ th receptor ${V}^{a}=\left\{{s}_{1}^{a},{s}_{2}^{a},{s}_{3}^{a},\dots ,{s}_{{N}_{mol}}^{a}\right\}$ is similar to that for the $b$ th receptor ${V}^{b}=\left\{{s}_{1}^{b},{s}_{2}^{b},{s}_{3}^{b},\dots ,{s}_{{N}_{mol}}^{b}\right\}$, where ${N}_{mol}$ is the number of ligand molecules in the interaction table. Thus, the ensemble of the vectors can be used for clustering the receptor ligand-binding sites. Similarly, if the structures of the $i$ th and $j$ th ligands are similar, the vector for the $i$ th ligand molecule ${U}_{i}=\left\{{s}_{i}^{1},{s}_{i}^{2},{s}_{i}^{3},\dots ,{s}_{i}^{{N}_{rec}}\right\}$ is similar to that for the $j$ th receptor ${U}_{j}=\left\{{s}_{j}^{1},{s}_{j}^{2},{s}_{j}^{3},\dots ,{s}_{j}^{{N}_{rec}}\right\}$, where ${N}_{rec}$ is the number of receptors in the interaction table. Similarity of ligand molecules is useful for the ligand-based in silico drug screening (Fukunishi et al. 2005, 2006c).

## Descriptors for docking poses: pharmacogram method and paring propensity of substructures

The docking poses are useful descriptors. The simplest 1D descriptor of docking pose is a SIFt vector. The original SIFt is a digitalized amino-acid sequence of the receptor in that the residues contacting to the ligand are set to 1 and the other residues to 0. There are many variations based on the original SIFt vector (Deng et al. 2004).

**Fig. 5:**
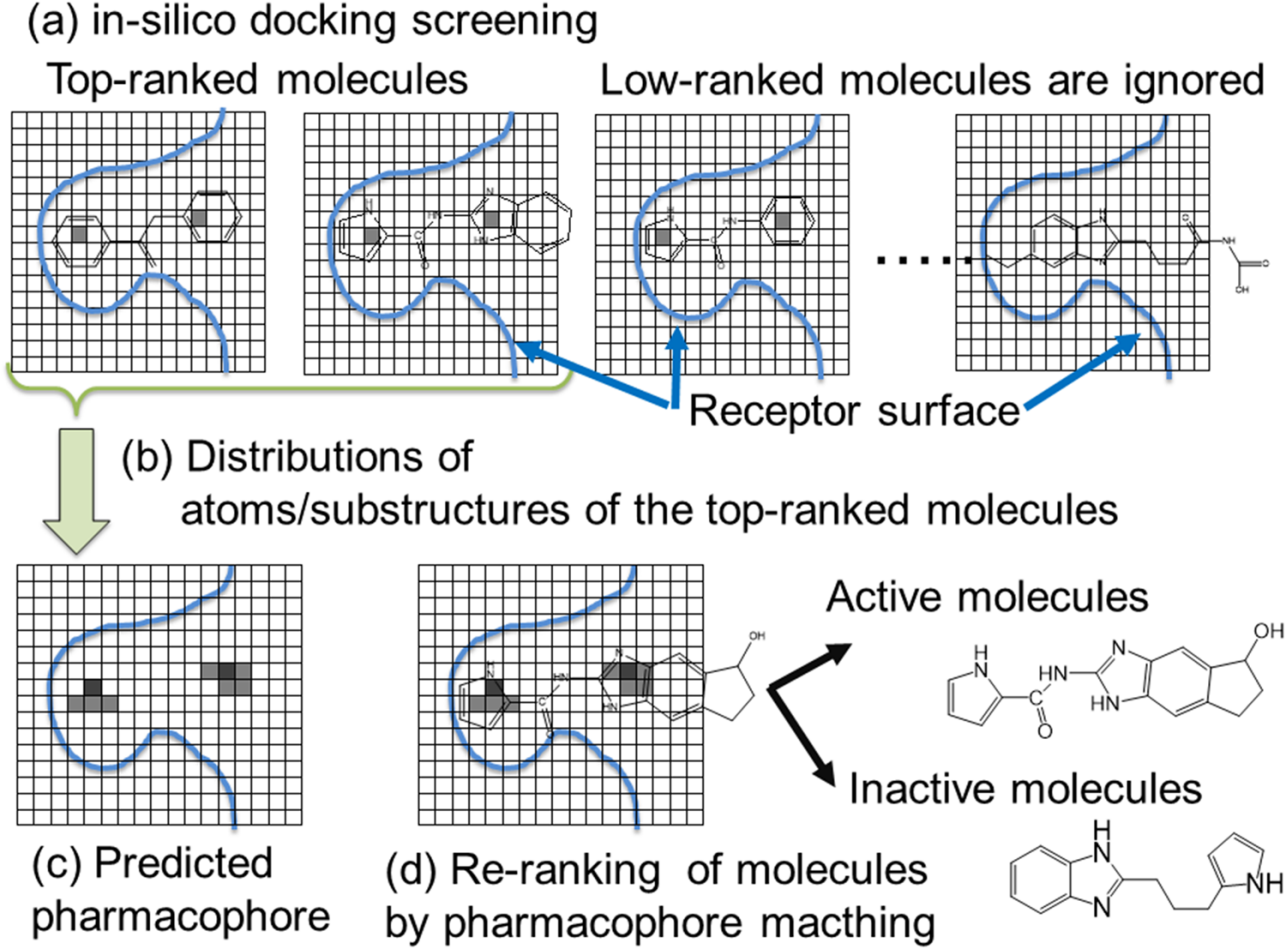
Schematic representation of the pharmacogram method and the grid-type descriptor of docking pose. Although the grids are presented three-dimensionally originally, this figure is presented two-dimensionally

Fujita and Orita introduced a 4D grid or multi-color 3D grid descriptor to represent the docking pose (see Fig. 5) and developed an in silico drug screening method called “pharmacogram method” (Fujita and Orita 2008). Receptor–ligand binding depends on the receptor-specific pharmacophore. “Pharmacophore” is a spatial distribution of steric and electronic features that contribute mainly to the optimal receptor–ligand interactions. The docking screening methods sort the ligand molecules by their docking scores, and the higher-ranked molecules are rich of actual active molecules rather than the lower-ranked molecules in many cases. The docking poses of the top-ranked molecules are likely to have important receptor–ligand interactions that could be a part of the pharmacophore. Thus, we can predict the pharmacophore based on the docking screening.

Figure 5 illustrates the procedure of the pharmacogram method. The receptor structures are set to the same position. We put the receptor-ligand complex structure in the 3D grid box $G$ and the ligand-binding site is put at the center of the box. The box $G$ consists of sub cells that are divided by the grids and the box $G$ is described as a matrix $G$ ($=G\left(m,i,j,k\right)$ where $m$, $i$, $j$ and $k$ are integers: $i$, $j$ and $k$ specify the position of sub cell, and $m$ does the type of atom involved in the grid). Here $G\left(m,i,j,k\right)=1$ and $G\left(m,i,j,k\right)=0$ mean that the *m*th atom type (or substructure) exists in the sub-cell $(i, j,k)$ and not, respectively. The indexes of sub cell (i, j, k) correspond to the (x, y, z) coordinates in the Cartesian space.

We make the matrix G for each docking pose of the top-ranked molecules of the docking screening (Fig. 5a, b). And the average G matrix of these top-ranked molecules should give the pharmacophore of the target receptor (see Fig. 5c). Finally, the docking poses are evaluated and re-ranked to show how much the obtained pharmacophore is satisfied (Fig. 5d).

Most of the current deep-learning type docking methods adopt the pharmacogram type (Ragoza et al. 2017) or GOLD type pose descriptors (Pereira et al. 2016) to scoring the docking poses.

## Prediction of ligand-binding sites of receptors based on the interaction table

Because the ligand-binding sites of enzymes and receptors are at a concave and hydrophobic, the sequences are conserved and the ligand-binding propensity of amino-acid residues at the ligand-binding sites shows clear trends mostly. An aromatic large residue is likely to bind to the ligand, although small residues are not: The trend of ligand-binding propensity is Trp > Phe > Tyr > His > Arg > … > Gly (Soga et al. 2007). Most of the ligand-binding site (pocket) prediction methods show high prediction accuracy by using these steric features and the amino-acid sequence information.

The conservation of amino-acid sequences and the 3D receptor–ligand complex structures in PDB suggest that the pocket shapes are classified into a limited number of shapes (so-called “pocketome”) (Kufareva et al. 2012). The PoSSuM database summarized pairs of the receptor’s pockets and their ligands (Ito et al. 2012).

Receptor–ligand docking should find the ligand-binding sites of the target receptor, to which native-ligand-like molecules binds. As mentioned in section “Docking screening for choosing target-selective molecules based on the interaction table,” a wide variety of molecules can bind to receptors, regardless of binding energy. The MolSite method replaces the various receptors in Fig. 4a by the various sites of the target receptor described in Fig. 4b. Then, the Molsite method performs receptor–ligand docking of a set of small compounds including small drug molecules to the various sites of the receptor surface and predicts the site that exhibits the strongest docking scores (Fukunishi and Nakamura 2011).

## Ensemble receptor–ligand docking

Structural dynamics are essential to realize the functions of enzymes and receptors. Namely, the ligand-binding sites change the structures during the ligand association and dissociation. These dynamics include the population shift, induced fit, local folding (coupled folding and binding), and so on. The definition of $\Delta G$ (Eq. 12) suggests that the docking results obtained from multiple receptor structures are closer to the reality than that from a single receptor structure.

Ensemble docking is a procedure where one ligand molecule binds to an ensemble of multiple receptor conformers to improve the accuracies of docking pose and binding activity prediction. Figure 4c shows the interaction matrix for the ensemble-docking screening. The ensemble of receptor structures can be obtained from various MD simulations such as the conventional MD simulation of receptor, generalized ensemble simulation, co-solvent MD simulations, and experiments (X-ray crystallography, liquid NMR, cryo-EM etc.).

The ensemble docking was considered when Kuntz’s group developed the first docking program DOCK (Kuntz et al. 1982; Meng et al. 1992; Ferrari et al. 2004). The early ensemble docking replaced the grid potential from a single receptor structure by an average over multiple grid potentials of receptor structures, and then the docking using the averaged grid potential was performed. This method did not increase the docking calculation cost. Currently, the ensemble-docking score is computed from the Boltzmann-weighted or simple average, and each docking score is obtained from the grid potential of each receptor structure of the ensemble (Knegtel et al. 1997).

A problem of ensemble docking is how to select the most suitable receptor structures from many structures in the ensemble since the in silico screening of millions or billion compounds are time consuming (Mohammadi et al. 2022). However, it is likely that a small number of receptor–ligand complex structures with strong binding energies contribute to a major part of the $\Delta G$. The key point is a careful structural clustering of receptor conformers to decrease the number of candidate structures when some experimental active ligand molecules are available: When such active molecules are available, machine learning methods (i.e., random forest, naïve Bayesian model, deep learning) can make a rule for selection.

Molsite is also useful for the ensemble docking (Fukunishi et al. 2010). The Molsite method predicts receptor’s surface sites that are likely to bind to ligand-like and drug-like molecules. Then, the predicted receptor sites are replaced by the receptor conformers in the ensemble. Suitable conformers are elected from conformers with high docking scores.

## Remained problems: cryptic site

Each cell expresses several thousands of genes and many proteins produced by those genes are crowded in the cell. These proteins may interact randomly and conflict mutually (crowding effect). In this situation, exposed hydrophobic surfaces of proteins may cause non-selective protein–protein bindings. To avoid such bindings, the surfaces of the proteins are almost hydrophilic. Recently, binding sites that are exposed only when binding to a ligand or that appear transiently in an apo form have been investigated (Cimermancic et al. 2016; Beglov et al. 2018; Vajda et al. 2018). Such binding sites are called “cryptic sites” and may be one of mechanics of forming functional protein–protein complex structures like transcription factor complexes (Bekker et al. 2021b; Iida et al. 2020).

The conventional pocket prediction methods were not so useful to find the cryptic sites with using an apo form of a receptor. On the other hands, since the cryptic sites are functional, the amino-acid sequences around cryptic sites are conserved, and MD simulations show that the cryptic sites are transiently appear in 100–1000 ns at a room temperature (Frembgen-Kesner and Elcock 2006; Guo et al. 2016).

Iida et al. found that the ligand-binding propensity of amino-acid at the cryptic site is different from that of the conventional ligand-binding site. Namely, Tyr and Phe are the most popular in the cryptic site, although Trp is the most popular in the conventional ligand-binding site (Iida et al. 2020). With analyzing PDB statistically and using information from MD simulations, they proposed a “cryptic-site index” that provides the propensity of each amino-acid to be in the cryptic site. The cryptic site index showed that the aromatic residues (Tye > Phe > His) except Trp tend to be in the cryptic sites. In many cases, several 100 ns MD simulations at the room temperature are enough to find the cryptic sites at positions predicted by the cryptic-site index values. Some previous works showed that the chance of opening the cryptic sites increases with increasing the vdW interaction between the solvent water molecules and receptor atoms (SWISH method) (Oleinikovas et al. 2016). The combination of co-solvent MD, the cryptic site index, and ensemble docking may make the drug screening effective when the ligand-binding site is the cryptic site.

## Enhanced sampling methods and molecular binding

Energy basins distribute in the conformational space and energy barriers hinder the inter-basin conformational transitions. As mentioned, when ensemble docking does not work because of the large conformational deformations/fluctuations of biomolecules during the complex formation, a powerful sampling method is required. One way is to use an MD-specialized computer such as ANTON (Shaw et al. 2008; Shaw et al. 2014) or MDGRAPE (Ohmura et al. 2014), MD Engine (Toyoda et al. 1999), or Express5800/MD server (Ohtaki et al. 2008). The other is to use an enhanced sampling (generalized ensemble) algorithm. In this review, we explain the latter because anyone can use a general-purpose computer.

To increase the sampling efficiency by algorithm, a generalized ensemble method such as a multicanonical method or a replica exchange method was proposed (Higo et al. 2012). The multicanonical algorithm was proposed first to study a physical system, a spin system, with using a MC simulation (Berg and Neuhaus 1992), applied to conformational motions of a biological system (Hansmann & Okamoto 1993; Kidera 1995), and incorporated in MD (Hansmann et al. 1996; Nakajima et al. 1997; Bartels and Karplus 1998). Similarly, the replica-exchange algorithm was developed to study a spin system using MC (Hukushima and Nemoto 1996) and applied to a biological system with using MD (Sugita and Okamoto 1999). Around the same time, several sampling methods, which have some similarity with the multicanonical or replica exchange methods, have been developed (Torrie and Valleau 1977; Paine and Scheraga 1985; Swendsen and Wang 1986; Mezei 1987; Lee 1993; Fukunishi et al. 1996; Iba et al. 1998; Wang and Landau 2001; Darve and Pohorille 2001; Laio and Parrinello 2002; Fukunishi et al. 2002; Hamelberg et al. 2004; Deng and Roux 2009; Moritsugu et al. 2010; Itoh and Okumura 2013; Peter and Shea 2014; Dasgupta et al. 2016; Kasahara et al. 2018; Ekimoto and Ikeguchi 2018; Higo et al. 2020b).

The enhanced sampling has a high efficiency to overcome energy barriers and importantly the method can assign a statistical weight equilibrated at a physiological temperature to any snapshot. Therefore, the resultant ensemble is equivalent to an equilibrated ensemble (canonical ensemble). By clustering the snapshots, one can identify basins in the conformational space, which means that Eq. 7 is computable. Suppose that the conformational space consists of three basins ${b}_{1}$, ${b}_{2}$, and ${b}_{3}$. Then, the free-energy ratio of ${F}_{{b}_{1}}$ and ${F}_{{b}_{2}}$ is expressed as:

20 $$\frac{{F}_{{b}_{1}}}{{F}_{{b}_{2}}}=\frac{\sum_{i}^{{n}_{1}}{w}_{i}^{{b}_{1}}}{\sum_{i}^{{n}_{2}}{w}_{i}^{{b}_{2}}} ,$$

where ${w}_{i}^{{b}_{j}}$ and ${n}_{j}$ were defined in Eq. 7. The normalization factor, which was omitted in Eq. 1, is cancelled out in Eq. 20.

Suppose that basin ${b}_{3}$ was sampled insufficiently (or not sampled at all). Even so, the ratio $\frac{{F}_{{b}_{1}}}{{F}_{{b}_{2}}}$ is computable correctly if ${b}_{1}$ and ${b}_{2}$ are sampled sufficiently. However, $\frac{{F}_{{b}_{3}}}{{F}_{{b}_{j}}}$ ($j=\mathrm{1,2}$) is computed inaccurately because of the insufficient data of ${b}_{3}$. Importantly, one may not notice this inaccuracy even after the simulation has finished. We note that such insufficiency occurs usually in minor basins fortunately. However, if the basin is a major one, main results from the sampling become misleading.

In enhanced sampling, a single or multiple reaction coordinates are introduced, which can be energy, temperature, Hamiltonian, other structural parameters (such as inter-molecular distance or radius of gyration), or a virtual quantity for instance. In brief, the sampling is enhanced along the reaction coordinates by adding a bias potential along the reaction-coordinate axes or by controlling transition probability between different reaction-coordinate positions. The variation of the reaction coordinate(s) can be either continuous or discrete.

Application of the enhanced sampling to molecular binding is increasing (Sinko et al. 2013). Two-dimensional (temperature and Hamiltonian) replica exchange sampling was combined to the Rosetta docking (Zhang et al. 2015). The replica-exchange method was applied to poses obtained from Rosetta to detect stable complex conformations (poses) (Wang et al. 2017). To develop a drug for SARS-CoV-2, the in silico screening followed by MD simulation was applied to many existing drugs, and then metadynamics was applied to remaining poses to select better drug candidates (Kumawat et al. 2021; Namsani et al. 2021). Binding poses from ensemble docking were assessed by metadynamics to screen out false positives (Dandekar et al. 2021). However, it is still difficult to apply the enhanced sampling to many systems because this method requires a long computation time. Even so, enhanced sampling is useful to obtain details of the molecular binding process. Amyloid aggregation process was investigated by a replica-permutation method (Itoh and Okumura 2021). Metadynamics was applied to a protein–ligand binding phenomenon that accompanies an induced-fit conformational change (Zhao et al. 2021).

Nakamura and his coworkers introduced a generalized ensemble method, a multi-dimensional virtual-system coupled molecular dynamics (mD-VcMD) (Hayami et al. 2019), and then the Genetic Algorithm (GA) was incorporated to mD-VcMD, which was named “GA-mD-VcMD” (Higo et al. 2020b). In this method, the entire multidimensional reaction-coordinate space is divided into many small pieces (zones). The conformation (phase point) moves freely only in a zone for a while, and occasionally the phase point transitions to another zone using an inter-zone transition probability, which is defined by a user. This method was applied to some biological systems to elucidate ligand-receptor binding mechanisms and produce free-energy landscapes (Higo et al. 2019, 2020a, 2021; Hayami et al. 2021).

**Fig. 6:**
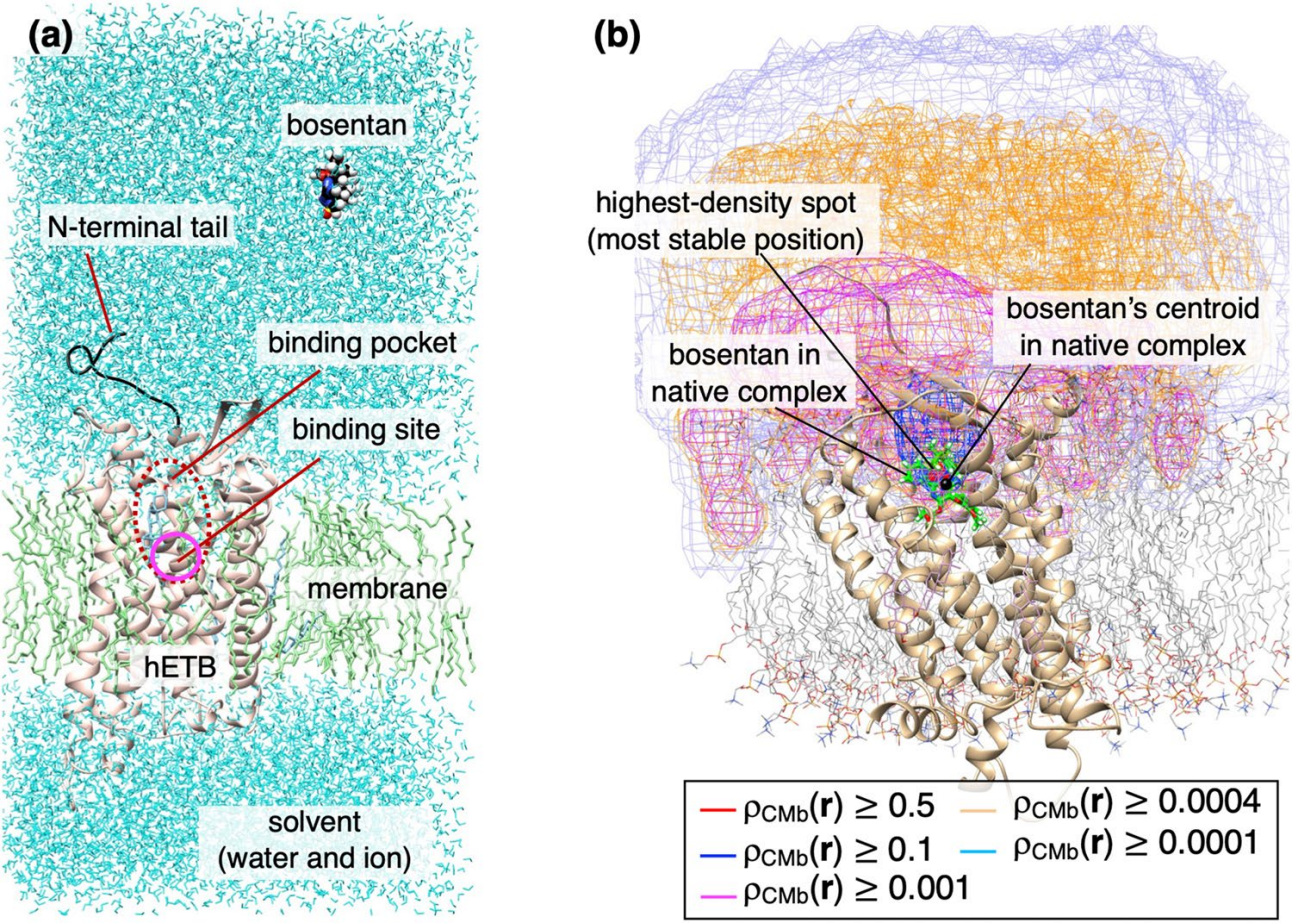
**a** Initial conformation of simulation consisting of hETB, bosentan, membrane (cholesterol and POPC lipid molecules), and solvent (water molecules and ions). All molecules are flexible. **b** Spatial density of bosentan’s centroid ${\rho }_{CMb}({\varvec{r}})$ at position ${\varvec{r}}$. Iso-density surface is presented by five differently colored contours (see inset). Green-colored stick model and black sphere are, respectively, bosentan and the bosentan’s centroid position in the native complex experimentally determined (PDB ID: 5xpr)

Here we introduce a study of GA-mD-VcMD applied to a middle-sized flexible drug, bosentan, binding to a GPCR molecule, human endothelin receptor type B (hETB) (Higo et al. 2022). Figure 6a illustrates the initial conformation of the simulation, where bosentan is far from hETB. The hRTB has a long N-terminal tail fluctuating largely in solution, and the root of the tail is located near the entrance of the gate of the binding pocket. The binding site is at the bottom of the pocket. Figure 6b demonstrates the resultant spatial density, ${\rho }_{MCb}({\varvec{r}})$, of the bosentan’s centroid at position ${\varvec{r}}$. The density was normalized so that ${\rho }_{CMb}({\varvec{r}})$ at the highest density position is 1.0. Apparently, the highest-density spot (region with ${\rho }_{CMb}\ge 0.5$; red-colored contours) corresponded to the bosentan’s position in the native complex (crystal structure) (Shihoya et al. 2017). The density was still high in the binding pocket (${\rho }_{CMb}\ge 0.1$). Although the density decreased with the ligand being apart from the binding pocket, this figure indicates that hETB and membrane affected bosentan even in a region far from hETB (${\rho }_{CMb}\ge 0.0004$). Subsequent analyses showed that this long-range effect is caused by contacts of bosentan to the N-terminal tail of hETB.

**Fig. 7:**
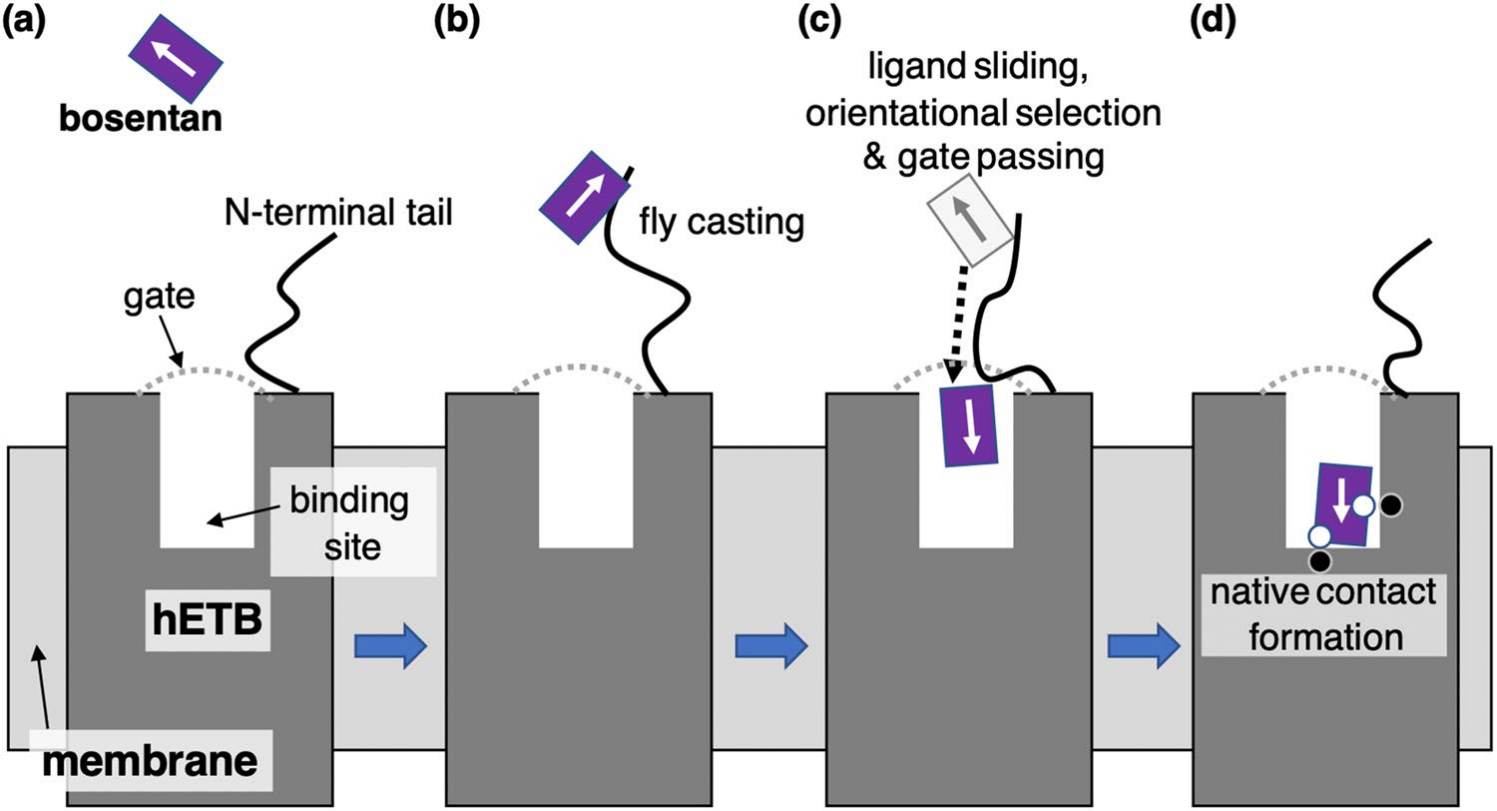
Bosentan-hETB binding process follows panels as $\left({\varvec{a}}\right)\to ({\varvec{b}})\to ({\varvec{c}})\to ({\varvec{d}})$. Arrow assigned to bosentan indicates its molecular orientation. Gate of the binding pocket is shown by gray dotted line. Ligand binding site is at the bottom of the binding pocket. Native attractive contacts are shown by pairs of open and filled small spheres in panel $({\varvec{d}})$

The binding mechanism of this system is summarized as follows. First, bosentan and the N-terminal tail of hETB are fluctuating in solution (Fig. 7a). Then, the tip of the N-terminal tail of hETB captures bosemtan via nonspecific attractive interactions (Fig. 7b), which is called “fly casting” (Shoemaker et al. 2000; Sugase et al. 2007; Arai 2018). Next, bosentan slides occasionally from the tip to the root of the N-terminal tail (ligand–sliding) (Fig. 7c). During this sliding, bosentan passes the gate of the binding pocket, which accompanies rapid reduction of the molecular orientational variety of bosentan. This molecular orientational reduction, called a “orientational selection,” is categorized to the population selection (Bosshard 2001; James and Tawfik 2003; Yamane et al. 2010), and consequently molecular orientations suitable for moving in the binding pocket toward the binding site are selected. Furthermore, this gate passing corresponds to overcoming a free-energy barrier in a free-energy landscape. When bosentan has reached the bottom of the pocket, attractive inter-molecular contacts are formed (formation of native contacts), which is the most thermodynamically stable complex (Fig. 7d). Details for this mechanism is reported in the paper (Higo et al. 2022).

Although the enhanced sampling (generalized ensemble) methods can assign a statistical weight to snapshots as mentioned above, the sampling requires a long simulation to obtain data that guarantee statistics accurate enough. One can perform multiple short runs instead of the long simulation, where the runs are distributed widely in the conformational space (Higo et al. 2009; Ikebe et al. 2011). However, the number of runs should be large when the system is complicated. For instance, the bosentan–GPCR simulation mentioned above, we performed 2000 runs. Therefore, it is still difficult to perform the enhanced sampling for many computational researchers. We, however, believe that the applicability of the enhanced sampling methods increases because the computer power is increasing rapidly and steadily.

## Binding free energy along a pathway: local sampling

**Fig. 8:**
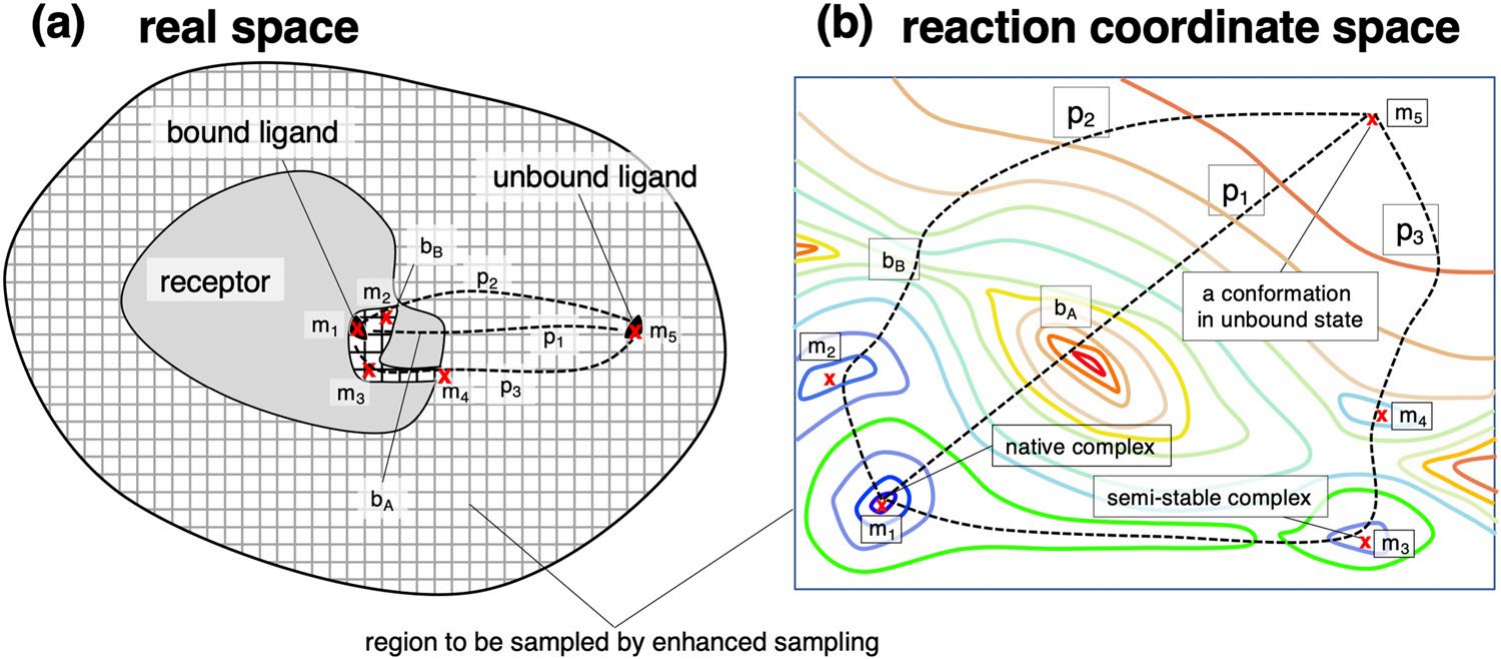
**a** Binding/dissociating pathways in real space. Although the space is three-dimensional, it is presented two-dimensionally. Red-colored “$\times$” labeled by ${m}_{i}$ ($i=1,\dots ,5$) is as follows: ${m}_{1}$ is the most stable position of ligand (native-complex position), ${m}_{2}$, ${m}_{3}$ and ${m}_{4}$ are semi-stable positions, and ${m}_{5}$ is a conformation in the unbound state. Ligands at ${m}_{1}$ and ${m}_{5}$ are shown as “bound ligand” and “unbound ligand,” respectively. Three ligand binding/dissociating pathways (${p}_{1}$, ${p}_{2}$ and ${p}_{3}$) are indicated by broken lines, which connect ${m}_{1}$ and ${m}_{5}$. Meshed area is region to be sampled by global sampling. Labels ${b}_{A}$ and ${b}_{B}$ indicates positions of energy barriers along ${p}_{1}$ and ${p}_{2}$, respectively. **b** Free-energy landscape in reaction-coordinate space. Blue to red contour lines correspond to low to high PMF values. Meaning of ${p}_{i}$, ${b}_{A}$, ${b}_{B}$, “$\times$” and ${m}_{i}$ are the same as those in panel **a**

As explained, the enhanced sampling method explores the conformational space widely with searching free-energy basins (binding poses). We refer to this approach as “global sampling” in this paper. The global sampling searches major basins to understand the binding process. Practically, on the other hand, the free-energy differences among the basins and the heights of free-energy barriers are not always estimated accurately when the computed system is large and complicated and when the simulation length is short. We suppose that the meshed area of Fig. 8a as well as the whole area of Fig. 8b are regions to be sampled by the global sampling.

If PMF is calculated along a pathway (line) in the real space, the volume to be sampled decreases drastically comparing with that sampled by the global sampling. We refer to this approach as a “line sampling,” which is an extreme case of local sampling. Of course, the line sampling cannot discover basins out of the pathway. However, when the two conformations are set from the native complex and an unbound conformation, this method is useful to estimate the binding free energy.

Figure 8a presents schematically three pathways ${p}_{1}$, ${p}_{2}$, and ${p}_{3}$, each of which connects the most stable ligand position ${m}_{1}$ (the native-complex position) and a position ${m}_{5}$ in the unbound state. Figure 8b is a free-energy (PMF) landscape presented in the reaction-coordinate space. Remember that PMF is a quantity assigned to a position ${\varvec{q}}$ (Eq. 6): $PMF=PMF({\varvec{q}})$. Then, the change of PMF from ${m}_{1}$ to ${m}_{5}$ is defined as $\Delta G=PMF\left({{\varvec{q}}}_{{\mathrm{m}}_{5}}\right)-PMF\left({{\varvec{q}}}_{{\mathrm{m}}_{1}}\right)$, where ${{\varvec{q}}}_{{\mathrm{m}}_{5}}$ and ${{\varvec{q}}}_{{\mathrm{m}}_{1}}$ are respectively the positions of ${m}_{1}$ and ${m}_{5}$ in Fig. 8b in the reaction-coordinate space. In theory, $\Delta G$ is independent of the pathway. We, however, note that $\Delta G$ does not equivalent to the binding free-energy (free-energy difference between the native complex state and the full unfolded state). The free energy of the native-complex state is contributed by many conformations in the native-complex basin around ${m}_{1}$ (Eq. 7). Similarly, the free energy of the unbound state is contributed by many conformations in the unbound state. Furthermore, the binding free energy is measured in a solution that contains many identical receptors and identical ligands. Therefore, some corrections should be applied to $\Delta G$. Wo do not explain the corrections in this paper. See a paper (Fukunishi 2009) for instance.

In fact, the free-energy profile was computed by a thermodynamic integration (Kirkwood 1935; Gelman and Meng 1998), a thermodynamic perturbation method (Zwanzig 1954; Beveridge and DiCapua 1989; Merz and Kollman 1989) or a weighted histogram analysis method (WHAM) (Kumar et al. 1992; Bartels 2000).

Practically, pathway setting is crucial to keep the accuracy of PMF in the line sampling. Problem of line sampling is that an appropriate pathway is unknown a priori. The pathway ${p}_{1}$ in Fig. 8a is simply set by a straight line between ${m}_{1}$ and ${m}_{5}$ in the real space. The corresponding pathway ${p}_{1}$ in the reaction-coordinate space is not necessarily straight, although it is straight in Fig. 8b. We prepared Fig. 8 so that a high energy barrier ${b}_{A}$ exists in ${p}_{1}$. Therefore, when the phase point is near ${b}_{A}$, a very strong force acts on the system, which causes a large numerical error in the resultant PMF. If the pathway is shifted slightly to a direction toward which the force decreases, then the numerical error decreases. By repeating this pathway resetting, the pathway may reach the pathway ${p}_{2}$ finally, because the barrier ${b}_{B}$ in ${p}_{2}$ corresponds to a saddle point of PMF along ${p}_{2}$ (Fig. 8b). Therefore, the pathway resetting will not provide the pathway ${p}_{3}$, which is the best pathway, along which no remarkable barriers exist.

Nakamura and his coworkers proposed a method to escape high energy barriers in setting the pathway (Fukunishi et al. 2003). This sampling method consists of iterative simulations. An iteration (say iteration $M$) starts from the last conformation of iteration $M-1$, and the sampling is limited around the initial conformation by applying a restraint potential around the initial conformation: Sampling is localized around the initial conformation (effective range for the restraint potential is given by user). Furthermore, a repulsive potential is added at the vicinity of conformations sampled during the iteration. Besides, the repulsive potential is usually a Gaussian centered at the sampled conformations. With proceeding the iteration, the repulsive potential is accumulated in a low potential-energy region, and this region is gradually eliminated from sampling. This means that the simulation trajectory is not trapped in the low potential-energy region. On the other hand, very instable (high potential energy) regions (barrier ${b}_{A}$ for instance) are also eliminated from sampling because of its high potential energy. When the next iteration (iteration $M+1$) is initiated, the repulsive potentials accumulated in iterations 1 to $M$ are used in iteration $M+1$ from the beginning. Thus, the conformation does not return to a stable region, which was sampled in iterations 1 to $M$. The first iteration usually starts from a stable conformation (the native complex structure) and sampling continues till the phase point reaches an unbound conformation. By repeating the iterations and connecting the generated trajectories by the WHAM (Kumar et al. 1992; Bartels 2000), one can obtained a line in the 3D space, which connect the bound conformation to the unbound conformation. This method, named a “filling potential” method, is a procedure to escape conformational trapping and detour around high energy regions.

Although the filling-potential method produces a binding pathway along which rapid energy changes do not occur, the pathway looks like a random-work trajectory involving winding or loop-like curves. This may cause an unnecessarily computation. Then, Nakamura and his coworkers proposed a method to smoothen the random-like pathway by connecting the initial and final conformations by a linear combination of Legendre polynomials: Smooth-reaction path generation (SRPG) method (Fukunishi et al. 2009).

The idea of the filling potential is categorized in a Taboo search (Fred 1986). Around the same time, similar sampling methods to the filling potential method were proposed: Local elevation (Huber et al. 1994), conformational flooding (Grubmüller 1995), Wang–Landau sampling (Wang and Landau 2001), metadynamics (Laio and Parrinello 2002), and accelerated molecular dynamics (Hamelberg et al. 2004).

As explained above, the line sampling cannot discover out-of-pathway basins. Contrarily, the global sampling requires a high computational cost although it can discover various basins. To compensate the drawbacks of the two approaches, Nakamura and his coworkers proposed a local sampling method (Bekker et al. 2017). First, a cylinder is set in the system so that it covers both the ligand-binding site of receptor and an unbound position of ligand in solvent. Then, a multicanonical MD simulation is performed within the cylinder to obtain a free-energy landscape. Next, a low free-energy pathway is set by connecting the native-complex state and an unbound conformation in the resultant landscape. Note that the cylinder can be replaced by a body of an arbitral shape to define an appropriate pathway.

This method saves a computational time because the sampling is restricted in a volume enough to define the appropriate pathway. This method was applied to some systems: A ligand cyclin-dependent kinase 2 binding to a aminopyrazole inhibitor, yielding a binding free-energy error of 0.5 kcal/mol to the experimental value (Bekker et al. 2017), a medium-sized ligand 3MR binding to β-secretase 1 (error of 0.4 kcal/mol) (Bekker et al. 2019), and a peptide (about 10 residues long) from the amyloid-β peptide binding to an antibody solanezumab (error of 1.3 kcal / mol) (Bekker et al. 2020b). This procedure was also used to predict appropriate binding poses of some systems: Inhibitor binding to the N-terminal domain of heat-shock protein 90 (Bekker et al. 2020a), the Asian-dominant allele human leukocyte antigen binding to an HIV-1 Nef protein epitope (Bekker and Kamiya 2021), antagonist alprenolol binding to a GPCR, β2-adrenergic receptor (Bekker et al. 2021a), and two medium-sized inhibitors (ABT-737 and WEHI-539) binding to the cryptic site of Bcl-xL (Bekker et al. 2021b).

## Conclusions

We reviewed various molecular binding methods from in silico screening to generalized ensemble methods. The in silico screening is a high throughput procedure because this method can provide binding poses of many ligand-receptor systems in a short time interval. This method is effective when the conformational change upon molecular binding is negligible in both the ligand and receptor. When a large conformational deformation occurs in receptor, ensemble docking becomes useful because the ensemble may involve the deformed conformation of the receptor. When both the ligand and receptor are deformed considerably upon binding, a generalized ensemble method (global sampling) is useful. This approach, however, requires a considerable computational time. The line sampling or local sampling are methods to reduce the computation cost and to focus on a restricted region essential for the molecular binding process.

Nakamura has been contributing to the life science databases (PDB, eF-site, HitPredict etc.) and biomolecular simulation algorithms for understanding the life systems. His works follow the “Algorithms + Data Structures = Programs” and the DIKW pyramid (Data, Information, Knowledge, and Wisdom hierarchy) (Wirth 1976; Rowley 2007). Many people know about the Wirth’s book and the DIKW Pyramid, but few spend their lives exploring it.
